# Complementarity of solution and solid state mechanochemical reaction conditions demonstrated by 1,2-debromination of tricyclic imides

**DOI:** 10.3762/bjoc.18.75

**Published:** 2022-06-24

**Authors:** Petar Štrbac, Davor Margetić

**Affiliations:** 1 Ruđer Bošković Institute, Bijenička cesta 54, HR-10002 Zagreb, Croatia

**Keywords:** ball milling, cycloaddition, debromination, Diels−Alder reaction, mechanochemistry

## Abstract

The solution phase 1,2-debromination of polycyclic imides using the Zn/Ag couple was successfully transferred to solid state mechanochemical conditions. The Zn/Ag couple was replaced by the Zn/Cu couple which was prepared without any metal activation by in situ ball milling of zinc and copper dusts. The advantage of the ball milling process is that the whole procedure is operationally very simplified. The reactive alkene generated was trapped in situ by several dienes and the respective Diels−Alder cycloadducts were obtained. It was demonstrated that mechanochemical milling offers complementary conditions to solution (thermal) reaction by allowing chemical transformations to proceed which were not possible in solution and vice versa.

## Introduction

The complementarity of reaction conditions [1–3] where the reaction takes place under some, but not under other conditions, or where a chemical reaction proceeds in a different way or mechanism is a useful feature in synthetic organic chemistry. Advantageously, more difficult substrates or limitations of the conditions can be overcome by the change of the reaction methods. One of the emerging synthetic methods is mechanochemistry [4–7], a greener alternative to carry out synthesis which complements heating, irradiation and electrochemistry as methods of chemical activation [8]. Based upon our experience in applications of this method to organic synthesis [9–12], we recognized its potential for the adjustment of conditions in zinc-mediated debromination reactions.

**Figure 1:**
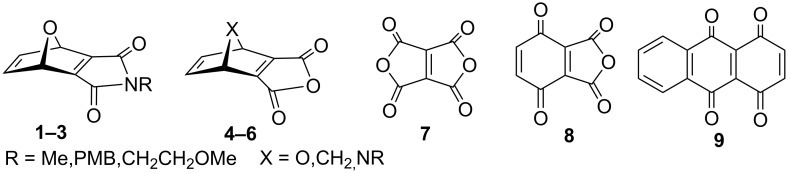
Highly reactive dienophiles.

Highly reactive dienophiles such as polycyclic molecules given in Figure 1 are interesting reactive intermediates which could be applied in the Diels−Alder reactions of less reactive or thermally susceptible dienes. Often, these are generated in situ and trapped with dienes in a single pot, such as 7-oxanorbornadiene imides **1–3**. For instance, a synthetic methodology for the preparation of **1–3** was developed by Warrener and co-workers by a Zn/Ag couple debromination [13–15]. However, this methodology has some disadvantages, such as tedious preparation of the catalyst, the use of dry solvent and expensive silver acetate as well as side-reactions with solvent.

An alternative method for the synthesis 7-oxanorbornadiene-2,3-anhydrides and imides is via retro Diels−Alder reaction employing the flash vacuum pyrolysis (FVP) technique [16]. However, the FVP also has disadvantages such as limited scope of functionalities which can withstand harsh conditions (temperature) and the inability to control the elimination process [17].

**Figure 2:**
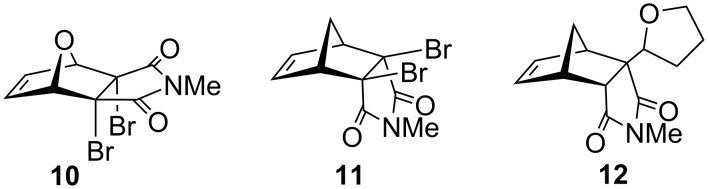
Dibromide substrates and product **12**.

The objective of this work was to establish whether the 1,2-debromination with the Zn/Ag couple could be carried out under solvent-free conditions in a ball mill and whether the tedious Zn/Ag couple preparation procedure [18] could be simplified by in situ generation of the catalyst. Moreover, in the debromination of norbornene imide **11**, the expected Diels−Alder adduct with furan was not obtained, but compound **12** incorporating a tetrahydrofuran ring at position 2, presumably by radical reaction (Figure 2) [19–20]. We envisaged that the absence of solvent under mechanochemical conditions should prevent the formation of products from tetrahydrofuran and therefore allow cycloaddition to take place.

## Results and Discussion

### Reaction optimization

**Scheme 1:**
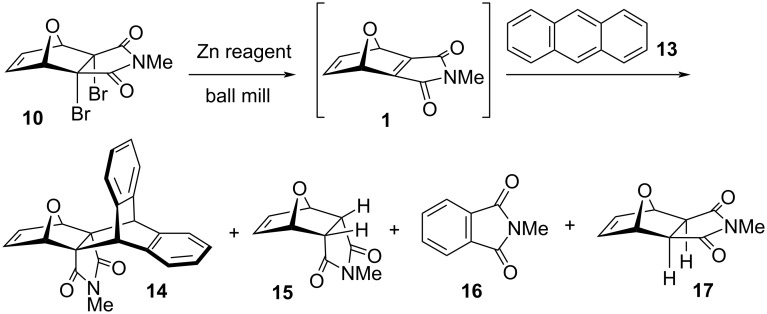
Mechanochemical reaction of **10** with anthracene.

Anthracene addition to dibromide **10** (Scheme 1) was used as the model reaction. Along with cycloadduct **14**, three known side-products were obtained: *endo-*product **15** (hydrogenolysed inverted **10**), *N*-methylphthalimide (**16**) and **17** (hydrogenolysed **10**). Their ratio varied with the reaction conditions (Supporting Information, Table S1) and results of the optimizations are collected in Table 1.

**Table 1:** Optimization of reaction conditions for reaction of **10** with anthracene.^a^

Entry	Catalyst	Additives	Time [h]	Conversion^b^	Yield **14** [%]^c^

1	Zn/Ag couple		0.5	81	2
2	Zn/Ag couple	NaCl	0.5	quant	8
3	Zn/Ag couple	LAG THF	0.5	quant	58
4	Zn/Ag couple	LAG THF, NaCl	0.5	96	33
5	Zn dust, Ag wire		0.5	NR^d^	
6	Zn dust, Ag wire	LAG THF	0.5	33	27
7	Zn dust, Ag wire	LAG MeOH	1	quant	64
8	Zn dust, Ag wire	LAG MeCN	1	88	63
9	Zn activ., Ag wire	LAG THF, NaCl	0.5	quant	56
10	Zn dust, silvergal		0,5	NR	
11	Zn dust, silvergal	LAG THF	1	50	40
12	Zn activated		0,5	NR	
13	Zn dust		0,5	NR	
14	Zn dust	LAG THF	0,5	12	4
15	Zn dust, Cu dust	LAG THF	0,5	55	50
16	Zn dust, Cu dust	LAG THF	0.75	97	64
17	Zn dust, Cu dust	LAG THF ZnBr_2_	1	quant	67
18	Zn dust, Cu dust	LAG THF^e^	1	66	48
**19**	**Zn dust, Cu dust**	**LAG THF** ^e^	**2**	**quant**	**76** (42)

	Reactions in solution	Solvent			

20	Zn/Ag couple	dry THF, Ar	1	quant	86
21	Zn dust, Cu dust	dry THF	1	NR	
22	Zn dust, Cu dust	THF, ultrasound	1.5	NR	

^a^Retsch MM400, 30 Hz, stainless steel 10 mL, one 12 mm SS ball; dibromide (50 mg); anthracene (132 mg, 5 equiv); reducing agent/catalyst (75 mg); silvergal = Ag/Cu powder 70% Ag; LAG THF η = 0.66 μL·mg^−1^; ^b^NMR analysis; ^c^NMR yields, isolated yield in parentheses; ^d^NR = no reaction; ^e^LAG THF η = 0.33 μL·mg^−1^.

The results of the optimization experiments showed that the solution reaction conditions could be transferred to mechanochemical conditions without significant loss of reactivity and identical side-products were formed. Initial experiments were performed with the Zn/Ag couple prepared by a usual procedure from Zn and silver acetate. Several simplifications of the Zn/Ag couple preparation were tested and showed that simple milling with Zn dust and Ag dust or wire can be also applied (Table 1, entries 5–8) [21]. Further improvement in the procedure was the replacement of the Ag dust with Cu dust. This combination of metals worked well, and the best conditions were obtained with the addition of a small amount of THF (liquid assisted grinding, LAG) [22], η = 0.5 μL·mg^−1^ (Table 1, entry 19). In contrast, the solution reaction catalyzed by Zn/Cu dust was totally ineffective (Table 1, entry 21), even with the agitation by ultrasound (Table 1, entry 22). The ratios of side-products vary depending on the reaction conditions (Table S1, Supporting Information File 1). The *endo*-product **15** was dominant in the neat grinding experiment (Table 1, entry 1), whereas phthalimide **16** dominates when NaCl was employed as grinding auxiliary (Table 1, entries 2 and 4). The addition of ZnBr_2_ (which is formed in the reaction and postulated that it could facilitate the oxa-ring opening of **15** to **16**) [13] did not notably increase the amount of phthalimide, indicating that rather deoxygenation leading to **16** is facilitated by the Zn/Cu couple [23]. When LAG THF reactions were carried out without anthracene, **15** was major product, whereas **16** is the major product in LAG MeOH milling (Supporting Information File 1, Table S1).

### Scope of the reaction

**Figure 3:**
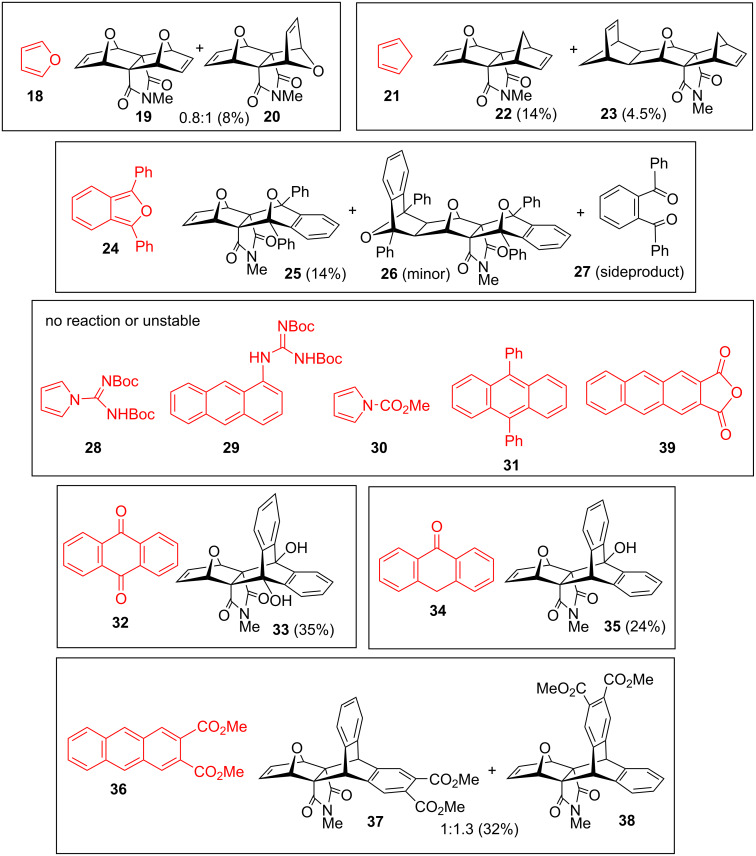
Scope of the Zn/Cu reaction with dibromide **10** (dienes are colored in red).

With the optimized conditions established, the scope of the reaction and its synthetic utility were investigated employing various dienes such as furan (**18**), 1,3-diphenylisobenzofuran (**24**) (DPIBF) and substituted anthracenes **31**, **36** and **39** (Figure 3). Exclusive norbornene *exo-*π selectivity [24] was observed in all cycloadddition reactions.

Selected five-membered dienes were subjected to established the Zn/Cu mechanochemical conditions (Figure 3). The furan reaction under ball milling conditions led to a mixture of *exo,exo-* and *exo,endo-*cycloadducts **19** and **20** in a 0.8:1 ratio. This is in contrast to classical conditions with the Zn/Ag couple in THF, where the ratio is different (0.6:1), and slightly more in favor of the unsymmetrical adduct **20**. On the other hand, the reaction of cyclopentadiene (**21**) provided the linear *exo,exo-*cycloadduct **22** as the major product, together with the 2:1 adduct **23**. In this reaction, identical stereospecificity was obtained by employment of the Zn/Ag couple in THF [19].

Linear *exo,exo-*product **25** was obtained exclusively in the reaction with DPIBF **24**, which is in accordance to the stereospecificity of cycloadditions reported by Sasaki [25] where the linear adduct is greatly preferred over bent. An interesting feature of the ^1^H NMR spectrum is the very low-field position of the phenyl protons (8.15 ppm), not common for DPIBF adducts with 7-oxanorbornenes (usually below 8 ppm) [26–27]. Double adduct **26** was detected as minor product (evidenced by the lack of olefinic and presence of the *endo* protons at 2.64 ppm), whereas the DPIBF sideproduct in this reaction is 1,2-dibenzoylbenzene (**27**). The mechanism for the formation of **27** from DPIBF is not elucidated, but we confirmed that the reaction does not proceed by milling of DPIBF with Zn/Cu dust alone, indicating that the presence of the dibromide substrate is essential. Product **27** was also found in the reaction of bicyclo[2.2.2] dibromide **42** (Scheme 3), which suggests that the mechanism may involve radical anion intermediates. This result further supports a single electron transfer (SET) and radical anion mechanism which was postulated earlier for the Zn/Ag debromination reaction and was supported by a CH_3_OD trapping experiment [13]. Thus, DPIBF in this reaction acts both as Diels−Alder trap reagent for reactive alkene [28] as well as radical anion quencher [29–30].

The *N,N*-Boc-protected *N*-amidinylpyrrole **28** [31] and 1-guanidinoanthracene (**29**) [10,32] have functional groups which were not tolerated under the debromination conditions and intractable mixtures of products were obtained. Carbomethoxypyrrole (**30**) was less susceptible to the reaction conditions, but unreactive. In these reactions, *N*-methylphthalimide (**16**) was obtained as the major product.

Interestingly, when 9,10-diphenylanthracene (**31**) was subjected to milling with **10**, the expected cycloadduct was not detected and **31** has remained unchanged, indicating its lower reactivity in comparison to anthracene (presumably due to steric reasons [33], the presence of phenyl substituents at reacting carbons). Instead, small amounts of another cycloadduct were obtained. It was found that this is the product arising from anthraquinone (**32**), which was present as an impurity in **31**. Independent milling of **10** with anthraquinone afforded dihydroxy cycloadduct **33** (in 35% yield) indicating that in the reaction conditions of the anthraquinone ↔ 9,10-dihydroxyanthracene (DHA) equilibrium is shifted towards DHA [34–35]. To prove this premise, anthraquinone alone was ball milled, however, unreacted material was recovered and the formation of 9,10-dihydroxyanthracene was not spectroscopically detected. An analogous hydroxy adduct **35** was produced in the reaction with anthrone (**34**). When the reaction of anthraquinone was carried in THF solution (reflux, 1 h), dibromide **10** remained unchanged. However, a small amount of **33** was formed in refluxing THF by the use of the Zn/Ag couple in the case of anthraquinone. These results indicate that the Zn/Cu catalyst in solid state is much more effective than Zn/Cu or Zn/Ag in THF solution, and that ball milling offers different reaction outcomes and as such complements solution chemistry.

The reaction with 2,3-dicarbomethoxyanthracene (**36**) afforded two isomeric cycloadducts **37** and **38** in a 1:1.3 ratio (inseparable mixture, 32% overall yield). The minor isomer has a linear structure with carbomethoxy groups in the equatorial plane as depicted for **37**, whereas in the major isomer **38** the carbomethoxy groups are positioned in axial plane of the molecule. The bent structure of **38** was established by ^1^H NMR analysis and comparison with product **10**. The most indicative signals are of *N*-methyl groups, which are at an almost identical position for the bent isomer **38** (2.36 ppm), as in products **10** and **33** (2.38 and 2.37 ppm, respectively). The chemical shift of the NMe group in linear product **36** is affected by anthracene carbomethoxy 2,3-substituents and is shifted towards lower magnetic field (2.42 ppm). Furthermore, the highest lying aromatic multiplet of **38** is similarly positioned as for **10** (7.06 and 7.08 ppm, respectively), whereas the chemical shift of the methyl ester groups in **38** is closer to the starting anthracene **36** (3.87 ppm and 3.85 ppm in **38** and **37** vs 3.97 ppm in **36**). When 2,3-anthracene anhydride (**39**) was subjected to ball milling, a complex mixture of products was obtained, indicating that the anhydride functionality is not compatible to the reaction conditions.

**Scheme 2:**
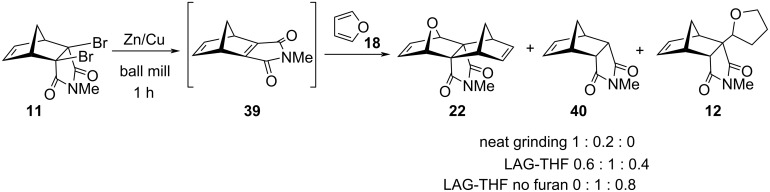
Mechanochemical reaction of **11** with furan.

Product **22** could be also obtained by cycloaddition parity reversal principle [16,36], employing norbornene dibromide **11** and furan (**18**). Ball milling reaction without THF (neat grinding) provided **22** as the major product, together with a minor amount of dehalogenated product **40** (Scheme 2). When THF was added for LAG, milling again gave **22**, but it was accompanied with a larger amount of **40** and some side-product **12**. A control LAG milling experiment without furan led to **40** and a significant amount of **12**. These experiments emphasize the complementarity of ball milling conditions with solution chemistry (in solution, only THF side-product **12** was obtained). Interestingly, small amounts of THF (used for LAG) were not detrimental for the reaction outcome in ball mill.

### The bicyclo[2.2.2] system

**Scheme 3:**
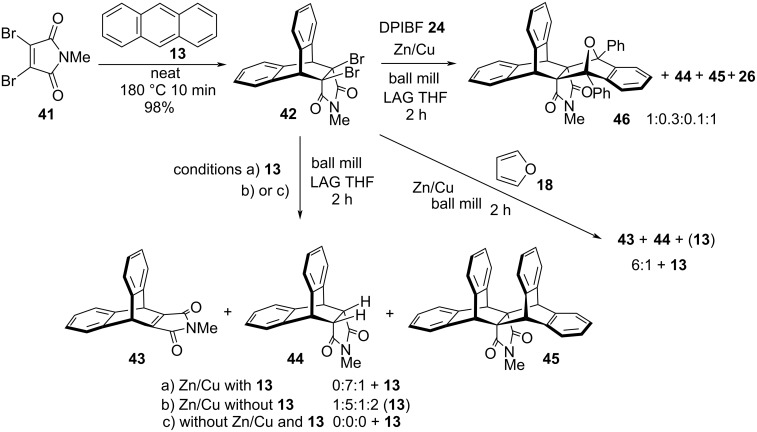
Reactivity of bicyclo[2.2.2] dibromide **42** with dienes.

Besides the bicyclo[2.2.1] system (7-oxa or 7-methano), the reactivity of the bicyclo[2.2.2] moiety was investigated. Dibromide **42** was prepared by Diels−Alder reaction of anthracene (**13**) and 2,3-dibromo-*N*-methylmaleimide (**41**) (Scheme 3). Heating of the reactants at 180 °C for 10 min provided the required cycloadduct **42** (98% yield). When **42** was subjected to Zn/Cu debromination in a ball mill in the presence of anthracene (conditions a), imide **44** was the major product accompanied by a small amount of janusene imide derivative **45** (7:1 ratio). The formation of the intermediate alkene **43** was observed spectroscopically only in the milling reaction of **42** alone (an indicative ^1^H NMR signal of bicyclo[2.2.1] moiety at 5.28 ppm). Cycloreversion side-reaction in mechanochemical conditions [37] was noticed for dibromide **42**, which led to mixtures consisting some janusene **45**. Thus, milling of **42** with Zn/Cu without anthracene (**13**) (conditions b) provided a mixture of **43**/**44**/**45**/anthracene in a 1:5:1:2 ratio) and milling of **42** alone without metal dust (conditions c) gave anthracene. Furthermore, alkene trapping with DPIBF provided 1:1 cycloadduct **46** as a single isomer (possessing linear structure as indicated by ^1^H NMR chemical shift of methyl group at 1.96 ppm). There is a large up-field shift of two aromatic protons of the anthracene moiety (6.31 ppm) arising from the magnetic shielding of phenyl groups. Analogously to the reaction of DPIBF with **10** (Figure 3), 1,2-dibenzoylbenzene (**27**) was found in the reaction mixture (Scheme 3). Starting from **42** in identical mechanochemical conditions, furan cycloadduct **14** was not formed, just **43** and **44** (6:1 ratio) and anthracene (**13**) were obtained. Our results demonstrate that the bicyclo[2.2.2] system is less reactive in comparison to the 7-oxabicyclo[2.2.1] system. Ball milling did not provide any cycloadduct even in the presence of 20-fold molar excess of furan and the alternative synthetic route (cycloaddition parity reversal) [16–17] for **14** was not viable.

## Conclusion

The 1,2-debromination reactions employing the Zn/Ag couple could be effectively carried out in a ball mill, with advantageous employment of the Zn/Cu couple prepared in situ, avoiding the use of dry solvent precautions and tedious Zn/Ag couple preparation, in a simple procedure. It is an example of organic reactions in solution that could be transferred to greener conditions. The reaction works in some instances when classical conditions failed, and as such it offers a complementary synthetic approach towards required molecules.

## Supporting Information

File 1 Details of experimental procedures and characterization data of selected compounds.
