# Supplementary material for: Complementarity of solution and solid state mechanochemical reaction conditions demonstrated by 1,2-debromination of tricyclic imides
Source: Beilstein J Org Chem. 2022 Jun 24;18:746–53. doi: 10.3762/bjoc.18.75 (PMC9235900; doi:10.3762/bjoc.18.75)
Supplement: File 1 — Details of experimental procedures and characterization data of selected compounds. [file Beilstein_J_Org_Chem-18-746-s001.pdf]

BEILSTEIN JOURNAL OF ORGANIC CHEMISTRY

Supporting Information

for

Complementarity of solution and solid state mechanochemical reaction conditions demonstrated by 1,2-debromination of tricyclic imides

Petar Štrbac and Davor Margetić

Beilstein J. Org. Chem. **2022,** *18,* 746–753. doi:10.3762/bjoc.18.75

Details of experimental procedures and characterization data of selected compounds

License and Terms: This is a supporting information file under the terms of the Creative Commons Attribution License (https://creativecommons.org/licenses/by/4.0). Please note that the reuse, redistribution and reproduction in particular requires that the author(s) and source are credited and that individual graphics may be subject to special legal provisions.
The license is subject to the *Beilstein Journal of Organic Chemistry* terms and conditions: (https://www.beilstein-journals.org/bjoc/terms)

1. **General** S1

2. **Synthetic details** S2

3. **NMR spectra** S8

4. **IR spectra** S21

5. **Table S1** S28

6. **References** S30

1. General

Chemicals and solvents were purchased from Sigma-Aldrich. Silvergal (dentistry silver copper amalgam, 70% Ag/Cu) was provided by Galenika. Kemika, Sigma Aldrich and VWR Chemicals supplied the solvents, which were used without further purification, unless otherwise stated. THF was dried over sodium/benzophenone.
All NMR spectra were recorded on Bruker Avance 300 MHz and Bruker Avance 600 MHz spectrometers in commercially available deuterated solvents. Chemical shifts (δ) are expressed in ppm according to tetramethylsilane (TMS) as internal standard, and coupling constants (*J*) are expressed in Hertz (Hz). The following abbreviations were used to denote multiplicity in ^{1}H spectra: s-singlet; d-doublet; dd-doublet of doublets; t-triplet; m-multiplet; brs-broad signal.
Infrared spectra (FTIR-ATR) were recorded using a Fourier transform infrared attenuated total reflection PerkinElmer UATR Two Spectrometer (from 400 cm^{-1} to 4000 cm^{-1}).
Milling reactions were carried out in a Retsch MM400 vibrational mill (frequency 30 Hz), using stainless steel (SS) vials (10 mL) and one 12 mm SS ball.
Thin layer chromatography was performed on silica gel plates (silica gel 60 F_{254}, Merck), whereas for column chromatography silica gel (Silica gel 60, 0.063-0.200 mm, Merck) was used. For each synthesis elution solvents and their ratio are specified.
High resolution mass spectra (HRMS) were recorded on a Waters Micromass Q-TOF micro and 4800 Plus Maldi TOF/TOF Analyser instrument.

2. Synthetic details

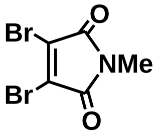

Preparation of *N*-methyl-2,3-dibromomaleimide (**41**)
A two-step literature procedure was followed.
Step 1) Preparation of 2,3-dibromomaleimide[1]
Succinimide (17 g, 172 mmol) was heated on an oil bath to 140 °C in a two neck 250 mL round-bottom flask fitted with a magnetic stirrer and reflux condenser. Bromine (26.6 mL) was added dropwise over 2.5 h to the molten succinimide while stirring. The temperature was held at 140–150 °C. Reaction mixture was cooled and 50 mL of water used to transfer the solid content of the flask to a beaker where it was digested for 2 hours at 60 °C with stirring. The solid was filtered off, washed well with water and dried. After drying the product was isolated as colorless solid (26.51 g, 60 %).
^{1}H NMR (DMSO-d_6) δ 11.65 (s, 1H, NH)
FTIR-ATR ν_{max}/cm^{-1}: 3224, 1722 (C=O), 1581, 1030.
Step 2) Methylation of 2,3-dibromomaleimide[2]
The 2,3-dibromomaleimide (26.0 g, 102 mmol), anhydrous potassium carbonate (16.0 g, 116 mmol) and dimethyl sulfate (10.5 mL, 111 mmol) were refluxed in acetone (130 mL) for two hours. The dark reaction mixture was poured into water (400 mL), left overnight and the product filtered off as buff colored crystals (12.1 g, 44%).
Spectral data are identical to literature.
^{1}H NMR ($CDCl_3$) δ 3.13 (s, 3H, CH_3)
FTIR-ATR ν_{max}/cm^{-1}: 2496, 1708 (C=O), 1679, 1385, 995.

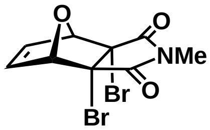

10

Preparation of cycloadduct **10**
The literature procedure was followed. A solution of *N*-methyl-2,3-dibromomaleimide (4.0 g, 14.9 mmol) in furan (8 mL) was heated at 70 °C over weekend in a sealed high pressure tube. After cooling solvent was removed in vacuum and solid was recrystallized from ethyl acetate to afford pure product as colorless solid (1.69 g, 34%).
Adduct **10** spectral data are identical to literature.[3]
^{1}H NMR ($CDCl_3$) δ 6.69 (t, *J*=1.1 Hz, 2H, C=CH), 5.34 (t, *J*=1.1 Hz, oxa-bridge), 3.09 (s, 3H, NMe)
FTIR-ATR ν_{max}/cm^{-1}: 3095, 1705 (C=O), 1428, 1380, 885.

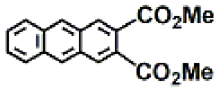

Preparation of 2,3-dicarbomethoxy anthracene (**36**)
The literature procedure was followed. To a solution of 2,3-anthracene dicarboxylic acid (3.72 g, 14 mmol) in methanol (150 mL) conc. sulfuric acid (3.5 mL) was added and stirred at reflux for 5 days. The mixture was then cooled to 0 °C, the solid was filtered, air dried and then

dissolved in mixture of water (80 mL) and CH_2Cl_2 (120 mL) with stirring. Separated CH_2Cl_2 layer was washed with 5% $NaHCO_3$ (20 mL), water (2 × 80 mL), dried over $MgSO_4$ and evaporated under vacuum to afford the pure product as yellow solid (0.850 g, 21%).
Anthracene **36** spectral data are identical to literature.[4]
^{1}H NMR ($CDCl_3$) δ 8.49 (s, 2H, Ar), 8.43 (s, 2H, Ar), 8.04 (dd, *J*=6.4, 3.2 Hz, 2H, Ar), 7.56 (dd, *J*=6.4, 3.2 Hz, 2H, Ar), 3.98 (s, 6H, Me).
FTIR-ATR ν_{max}/cm^{-1}: 2958, 1712 (C=O), 1294, 1228, 1048.

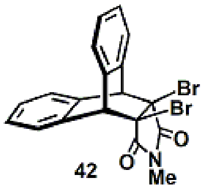

Adduct **42**
A mixture of anthracene (100 mg, 0.56 mmol) and *N*-methyldibromomaleimide (60 mg, 0.22 mmol) was heated in round bottomed flask attached to glass tube in pyrolysis furnace at 250 °C for 15 min. After cooling, excess anthracene was removed by sublimation in high vacuo to afford the product as colourless solid (100 mg, 98%).
^{1}H NMR ($CDCl_3$) δ 7.47 (dd, *J*=5.5, 3.5 Hz, 2H, Ar), 7.31 (dd, *J*=5.5, 3.5 Hz, 2H, Ar), 7.27 (dd, *J*=5.5, 3.5 Hz, 2H, Ar), 7.17 (dd, *J*=5.5, 3.5 Hz, 2H, Ar), 4.95 (s, 2H bridge-[2.2.2]), 2.59 (s, 3H, NMe).
^{13}C NMR ($CDCl_3$) δ 171.6 (C=O), 137.6, 137.1, 128.2, 127.5, 126.8, 125.7, 66.1, 54.9, 25.8 (CH_3).
FTIR-ATR ν_{max}/cm^{-1}: 2962, 1713 (C=O), 1016, 766.
HRMS-MALDI found: $[M+H]^+$: 445.9382. calculated for $C_{19}H_{13}N_1O_2Br_2$ 444.9313, $[M+H]^+$: 445.9391.

Preparation of the Zn/Ag couple [5]

Zinc dust (150 mg) was activated with 10% hydrochloric acid (0.70 mL), washed successively with acetone (2 × 1 mL) and diethyl ether (1 mL). A suspension of silver acetate (5 mg) in boiling acetic acid (0.70 mL) was then added, and the mixture stirred for 1 minute. The supernatant liquid was decanted and the zinc was washed with diethyl ether (4 × 1 mL) and then with methanol (1 mL).

General ball-milling procedure with Zn/Cu couple

Dibromide **10** (50 mg, 0.15 mmol), diene (5 equiv., excess), zinc dust (75 mg, 1.15 mmol, 7.7 equiv., excess), copper dust (15 mg 0.24 mmol, 1.6 equiv, excess) and THF (η = 0.33 or 0.66 μL mg^{-1}) were ball milled at 30 Hz for set time. Reaction mixture was then dissolved in a small volume of dichloromethane, and the solution was filtered through a short plug of Celite. The products were separated by radial chromatography and details are described for each product.

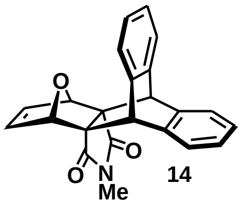

Adduct **14**
Purified by radial chromatography, elution solvent 10% ethyl acetate in petroleum ether. Colorless solid, isolated yield 42%.
^{1}H NMR ($CDCl_3$) δ 7.31 (dd, *J*=5.4, 3.5 Hz, 2H, Ar), 7.23 (dd, *J*=5.4, 3.5 Hz, 2H, Ar), 7.17 (dd, *J*=5.4, 3.5 Hz, 2H, Ar), 7.04 (dd, *J*=5.4, 3.5 Hz, 2H, Ar), 6.42 (s, 2H, C=CH), 4.80 (s, 2H, bridge-oxa), 4.74 (s, 2H, bridge [2.2.2]), 2.35 (s, 3H, NMe);
^{13}C NMR ($CDCl_3$) δ 176.7 (C=O), 141.2, 139.9, 137.9, 126.9, 126.8, 124.9, 124.4, 81.6 (oxa bridge), 65.5, 48.1 (bridge [2.2.2]), 24.0 (NMe),
FTIR-ATR ν_{max}/cm^{-1}: 2198, 1694 (C=O), 1289, 1015.
HRMS-MALDI found: $[M+H]^+$: 356.1290, calculated for $C_{23}H_{17}N_1O_3$ 355.1208, $[M+H]^+$: 356.1287.

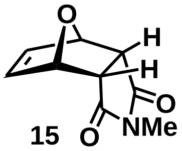

15

Product **15**
Spectral data obtained from crude spectra, are in accordance to literature [4]
^{1}H NMR ($CDCl_3$) δ 6.37 (t, *J*=1.1 Hz, 2H, C=CH), 5.29-5.32 (m, 2H, oxa-bridge), 3.50 (dd, *J*=3.5, 1.6 Hz, 2H, exo-H), 2.79 (s, 3H, NMe).

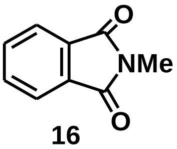

16

N-methylphthalimide **16**
NMR Spectral data are in accordance to literature [6]
^{1}H NMR ($CDCl_3$) δ 7.81 (dd, *J*=5.5, 3.0 Hz, 2H, ArH), 7.67 (dd, *J*=5.5, 3.0 Hz, 2H, ArH), 3.15 (s, 3H, NMe).
FTIR-ATR ν_{max}/cm^{-1}: 2924, 1706 (C=O), 1431, 1378, 1006.

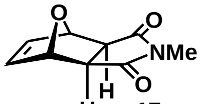

17

Product **17**
Spectral data obtained from crude spectra, are in accordance to literature [4]
^{1}H NMR ($CDCl_3$) δ 6.48 (t, *J*=1.1 Hz, 2H, C=CH), 5.25 (t, *J*=1.1 Hz, 2H, oxa-bridge), 2.96 (s, 3H, NMe), 2.84 (s, 2H, *endo*-H).

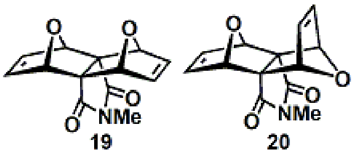

19 20

Adducts **19** and **20**
Purified by preparative TLC, elution solvent dichloromethane / 1% methanol, then polarity increased to 2% to afford a mixture of **19** and **20** as colorless solid (8% yield).
Spectral data obtained from mixture of two isomers are in accordance to literature [4]
Adduct **19**: ^{1}H NMR ($CDCl_3$) δ 6.56 (t, *J*=1.1 Hz, 4H, C=CH), 5.21 (t, *J*=1.1 Hz, 4H, oxa bridge), 2.67 (s, *J*=8.7, 3H, CH_3)

Adduct **20** : ^{1}H NMR (CDCl$_3$) δ 6.52 (t, *J*=0.9 Hz, 2H, =CH), 6.49 (t, *J*=0.9 Hz, 2H, C=CH), 5.12 (t, *J*=0.9 Hz, 2H, oxa bridge), 4.72 (t, *J*=0.9 Hz, 2H, oxa bridge), 2.84 (s, *J*=8.7, 3H, CH$_3$)

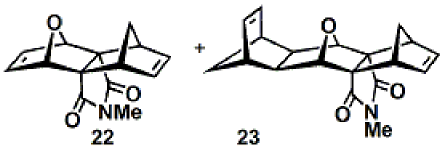

Adducts **22** and **23**
Purified by preparative TLC, elution solvent dichloromethane – 1% methanol, then the polar solvent was increased to 2%.
Adduct **22** colorless solid, (yield 14%).
^{1}H NMR spectral data are in accordance to literature [7]
^{1}H NMR (CDCl$_3$) δ 6.54 (t, *J*=0.9 Hz, 2H, C=CH oxa side), 6.31 (t, *J*=1.5 Hz, 2H, C=CH CH$_2$ side), 5.06 (t, *J*=0.7 Hz, 2H, bridge-oxa), 3.54 (nm, 2H, bridge CH$_2$), 3.06 (td, *J*=8.9, 1.2 Hz, 1H, CH$_2$), 2.69 (s, 3H, NMe), 1.69 (td, *J*=8.9, 1.2 Hz, 1H, CH$_2$).
^{13}C NMR (CDCl$_3$) δ 177.2 (C=O), 140.4, 138.9, 81.5 (oxa-bridge), 79.6, 68.8, 49.5, 46.1, 24.2 (CH$_3$).
FTIR-ATR ν_{max}/cm^{-1}: 2954, 1689 (C=O), 1427, 1285, 1018.
HRMS-MALDI found: [M+H]$^+$: 244.0982, calculated for $C_{14}H_{13}N_1O_3$ 243.0895 [M+H]$^+$: 244.0974.
Adduct **23** colorless solid (yield 4.5%).
^{1}H NMR (CDCl$_3$) δ 6.18 (t, *J*=1.3 Hz, 2H, C=CH), 5.97 (t, *J*=1.5 Hz, 2H, C=CH), 4.34 (s, 2H, bridge-oxa), 3.17 (t, 2H, *J*=1.2 Hz, bridge CH$_2$), 2.88 (brs, 2H, bridge CH$_2$), 2.87 (d, *J*=8.4 Hz, 1H, CH$_2$), 2.81 (s, 3H, NMe), 2.35 (dd, *J*=2.5, 1.4 Hz, 2H, *endo*), 1.39 (td, *J*=10.1, 1.2 Hz, 1H, CH$_2$), 1.33 (t,d *J*=8.2, 1.2 Hz, 1H, CH$_2$), 1.12 (d, *J*=8.4 Hz, 1H, CH$_2$).
FTIR-ATR ν_{max}/cm^{-1}: 2963, 1695 (C=O), 1284, 1021.

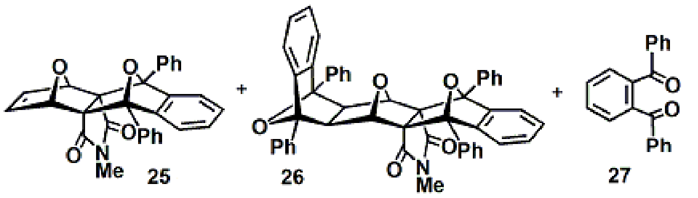

Adducts **25** and **26**
Products were purified by radial chromatography, elution solvent 1% methanol in dichloromethane.
Adduct **25** colorless solid (yield 14 %) ^{1}H NMR (CDCl$_3$) δ 8.15 (dd, *J*=8.0, 1.2 Hz, 4H, Ar), 7.59 (t, *J*=7.4 Hz, 4H, Ar), 7.49 (tt, *J*=7.4, 1.1 Hz, 2H, Ar), 7.12 (dd, *J*=5.5, 3.3 Hz, 2H, Ar), 7.07 (dd, *J*=5.5, 3.3 Hz, 2H, Ar), 6.55 (t, *J*=0.7 Hz, 2H, C=CH), 5.04 (t, *J*=0.7 Hz, 2H, bridge-oxa), 2.33 (s, 3H, NMe).
^{13}C NMR (CDCl$_3$) δ 196.1 (C=O), 139.5, 136.7, 132.5, 129.7, 129.3, 129.2, 127.8, 122.7, 80.6, (oxa-bridge), 76.2 (oxa-bridge), 56.2, 50.1 (CH$_3$).
FTIR-ATR ν_{max}/cm^{-1}: 2918, 1693 (C=O), 1289, 635.
HRMS-MALDI found: [M+Na]$^+$ 470.1396, calculated for $C_{29}H_{21}N_1O_4$: 447.1471. [M+H]$^+$: 448.1549, [M+Na]$^+$: 470.1368.
Adduct **26**
Obtained from crude spectra:

^{1}H NMR ($CDCl_3$) δ 8.20-6.94 (m, ArH overlap with impurities), 4.46 (s, 2H, bridge-oxa), 2.64 (s, 2H, *endo*), 2.31 (s, 3H, NMe).
1,2-dibenzoylbenzene **27**
Colorless solid, spectral data are in accordance to literature[8]
^{1}H NMR ($CDCl_3$) δ 7.69-7.71 (m, 4H, Ar), 7.61-7.62 (m, 4H, ArH), 7.49-7.53 (m, 2H, ArH), 7.36-7.39 (m, 4H, ArH),
^{13}C NMR ($CDCl_3$) δ 196.1 (C=O), 139.5, 136.7, 132.5, 129.9, 129.3, 129.2, 127.8.

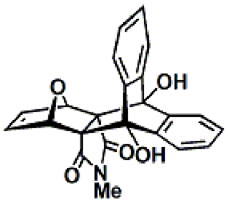

Adduct **33**
Purified by preparative TLC, elution solvent dichloromethane. Colorless solid (yield 35%).
^{1}H NMR ($CDCl_3$) δ 7.62 (dd, *J*=5.7, 3.3 Hz, 2H, Ar), 7.41 (dd, *J*=5.5, 3.3 Hz, 2H, Ar), 7.27 (dd, *J*=5.7, 3.3 Hz, 2H, Ar), 7.17 (dd, *J*=5.5, 3.3 Hz, 2H, Ar), 6.45 (t, *J*=0.9 Hz, 2H, C=CH), 5.12 (t, *J*=0.9 Hz, 2H, bridge-oxa), 4.24 (brs, 2H, OH), 2.37 (s, 3H, NMe).
^{13}C NMR ($CDCl_3$) δ 177.2 (C=O), 141.8, 138.4, 137.9, 127.0, 126.9, 120.3, 79.4 (oxa-bridge), 77.0, 68.4, 24.2 (CH_3).
FTIR-ATR ν_{max}/cm^{-1}: 2918, 1660 (C=O), 1447, 1272.
HRMS-MALDI found: 387.0318, calculated for $C_{23}H_{17}N_1O_5$ 387.1107 $[M+H]^+$: 388.1185.

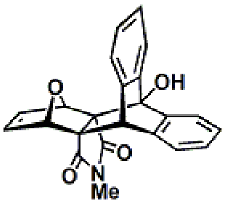

Adduct **35**
Purified by preparative TLC, elution solvent dichloromethane. Colorless solid (yield 24%).
^{1}H NMR ($CDCl_3$) δ 7.05-7.32 (m, 8H, Ar), 6.44 (d, *J*=5.6 Hz, 1H, C=CH), 6.42 (d, *J*=5.6 Hz, 1H, C=CH), 5.09 (s, 1H, bridge-oxa), 4.83 (s, 1H, bridge-oxa), 4.69 (s, 1H, bridge [2.2.2]), 4.37 (brs, 1H, OH), 2.36 (s, 3H, NMe).
^{13}C NMR ($CDCl_3$) δ 177.6 (C=O), 176.3 (C=O), 143.3, 140.4, 139.5, 137.9, 137.8, 137.7, 127.0, 126.96, 126.91, 126.87, 124.5, 123.8, 120.9, 120.7, 81.7 (oxa-bridge), 79.2 (oxa-bridge), 78.4 (q-OH), 66.8 (q), 66.6 (q), 46.9 (bridge [2.2.2]), 24.1 (CH_3).
FTIR-ATR ν_{max}/cm^{-1}: 3375, 2918, 1682 (C=O), 1018.
HRMS-MALDI found: $[M+H]^+$: 372.1240, calculated for $C_{23}H_{17}N_1O_4$ 371.1157 $[M+H]^+$: 372.1236.

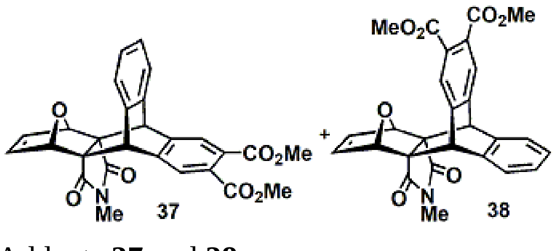

Adducts **37** and **38**

Purified by radial chromatography, elution solvent 1% methanol in dichloromethane. Colorless solid, mixture 1:1.3 (yield 32%). Spectral data were obtained from the mixture of two isomeric adducts.

Adduct **37**

^{1}H NMR ($CDCl_3$) δ 7.59 (s, 2H, Ar), 7.30 (dd, *J*=5.5, 3.3 Hz, 2H, Ar), 7.17 (dd, *J*=5.5, 3.3 Hz, 2H, Ar), 6.42 (s, 2H, C=CH), 4.81 (s, 4H, bridge-oxa, bridge [2.2.2]), 3.84 (s, 6H, CO_2Me), 2.42 (s, 3H, NMe).

Adduct **38**

^{1}H NMR ($CDCl_3$) δ 7.67 (s, 2H, Ar), 7.21 (dd, *J*=5.5, 3.3 Hz, 2H, Ar), 7.06 (dd, *J*=5.5, 3.3 Hz, 2H, Ar), 6.41 (s, 2H, C=CH), 4.83 (s, 2H, bridge-oxa), 4.81 (s, 2H, bridge [2.2.2]), 3.86 (s, 6H, CO_2Me), 2.36 (s, 3H, NMe).

HRMS-MALDI found: $[M+H]^+$: 472.1404, calculated for $C_{27}H_{21}N_1O_7$: 471.1318 $[M+H]^+$: 472.1396.

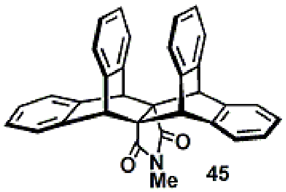

45

Adduct **45**

Spectral data obtained from crude spectra:

^{1}H NMR ($CDCl_3$) δ 7.47 (dd, *J*=5.5, 3.5 Hz, 2H, Ar), 7.31 (dd, *J*=5.5, 3.5 Hz, 2H, Ar), 7.27 (dd, *J*=5.5, 3.5 Hz, 2H, Ar), 7.17 (dd, *J*=5.5, 3.5 Hz, 2H, Ar), 4.95 (s, 2H bridge-[2.2.2]), 2.59 (s, 3H, NMe).

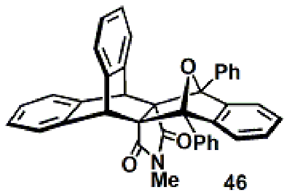

46

Adduct **46**

Purified by preparative TLC, elution solvent dichloromethane. Colorless solid (yield 77 %).

^{1}H NMR ($CDCl_3$) δ 7.89 (brs, 4H, Ar), 7.47-7.59 (m, 6H, Ar), 7.16 (dd, *J*=5.4, 3.1 Hz, 2H, Ar), 7.03 (brs, 4H, Ar), 6.97 (dd, *J*=5.4, 3.1 Hz, 2H, Ar), 6.90 (dd, *J*=5.1, 3.1 Hz, 2H, Ar), 6.32 (dd, *J*=5.4, 3.1 Hz, 2H, Ar), 4.64 (s, 2H bridge-[2.2.2]), 1.96 (s, 3H, NMe).

^{13}C NMR ($CDCl_3$) δ 176.3 (C=O), 139.3, 134.3, 127.6, 127.1, 126.6, 125.7, 124.9, 120.3, 90.2 (oxa bridge), 70.2 (bridge-[2.2.2]), 47.3, 23.8 (CH_3).

FTIR-ATR ν_{max}/cm^{-1}: 3023, 1698 (C=O), 1298, 703.

HRMS-MALDI found: 381.1365 $[M\text{-anthracene}+H_2]^+$: calculated for $C_{39}H_{27}N_1O_3$ 557.1991 $[M+H]^+$: 558.2069, $[M\text{-anthracene}+H_2]^+$: 381.1315.

3. NMR spectra

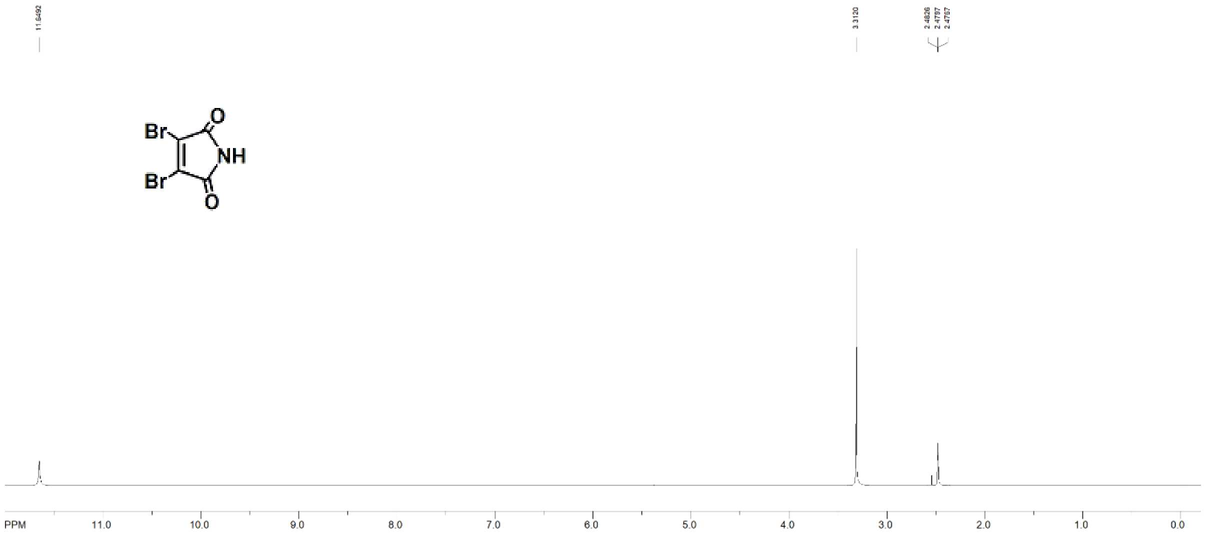

S1. ¹H NMR (300 MHz, $CDCl_3$) of 2,3-dibromomaleimide.

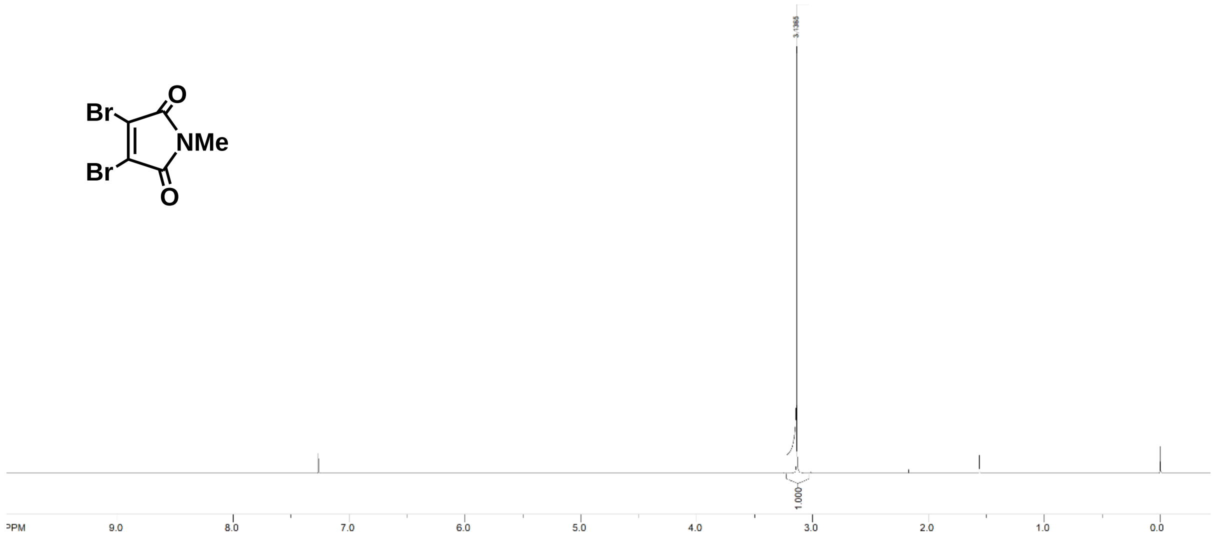

S2. ¹H NMR (300 MHz, $CDCl_3$) of *N*-methyl-dibromomaleimide **41**.

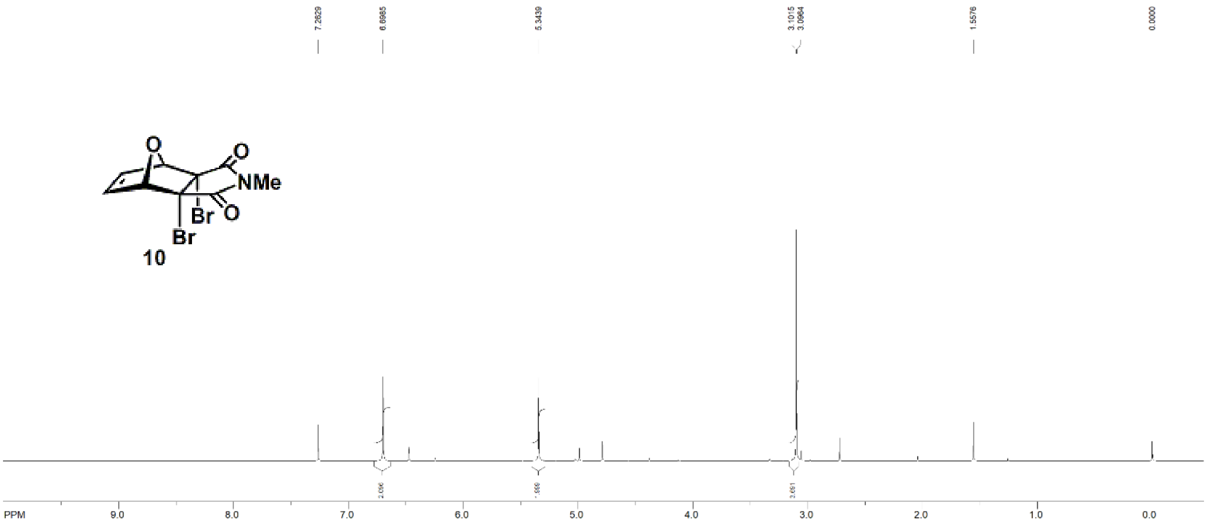

S3. ^{1}H NMR (300 MHz, $CDCl_3$) of cycloadduct **10**.

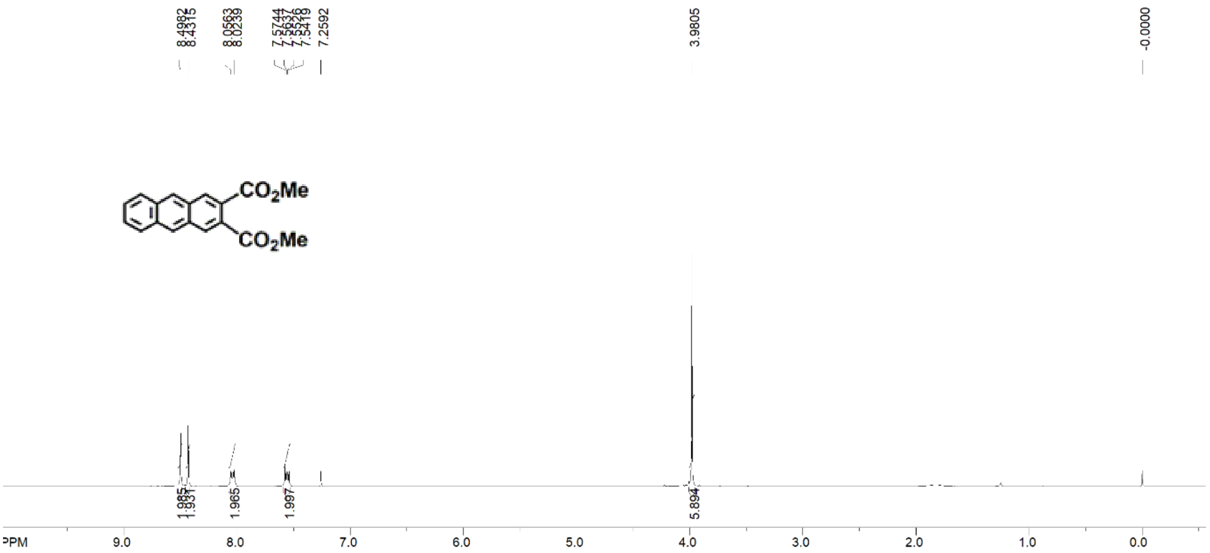

S4. ^{1}H NMR (300 MHz, $CDCl_3$) spectrum of anthracene **35**.

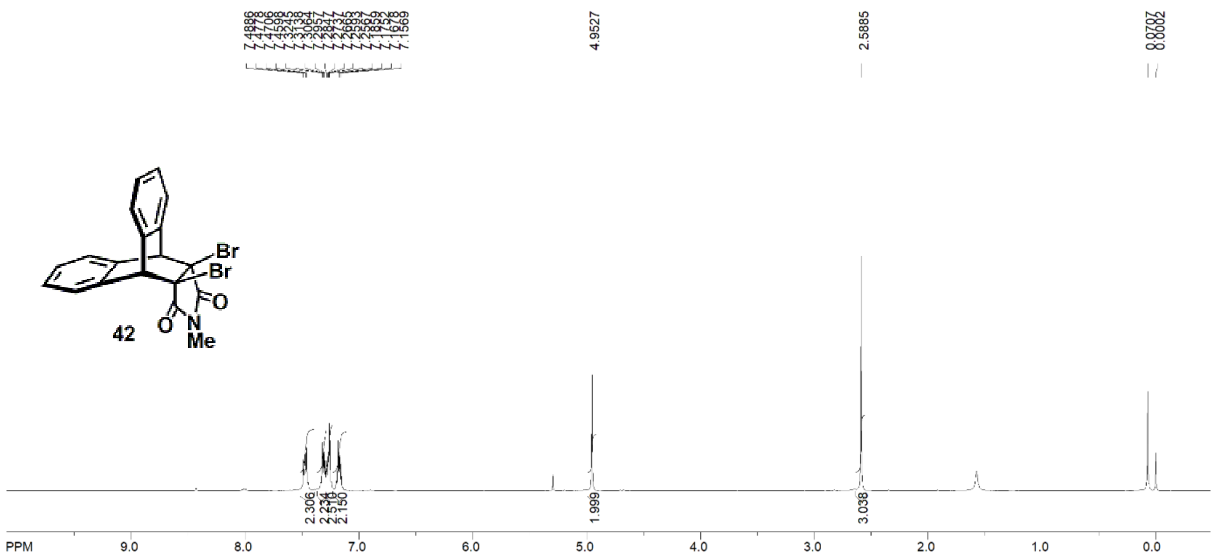

S5. ^{1}H NMR (300 MHz, $CDCl_3$) spectrum of **42**.

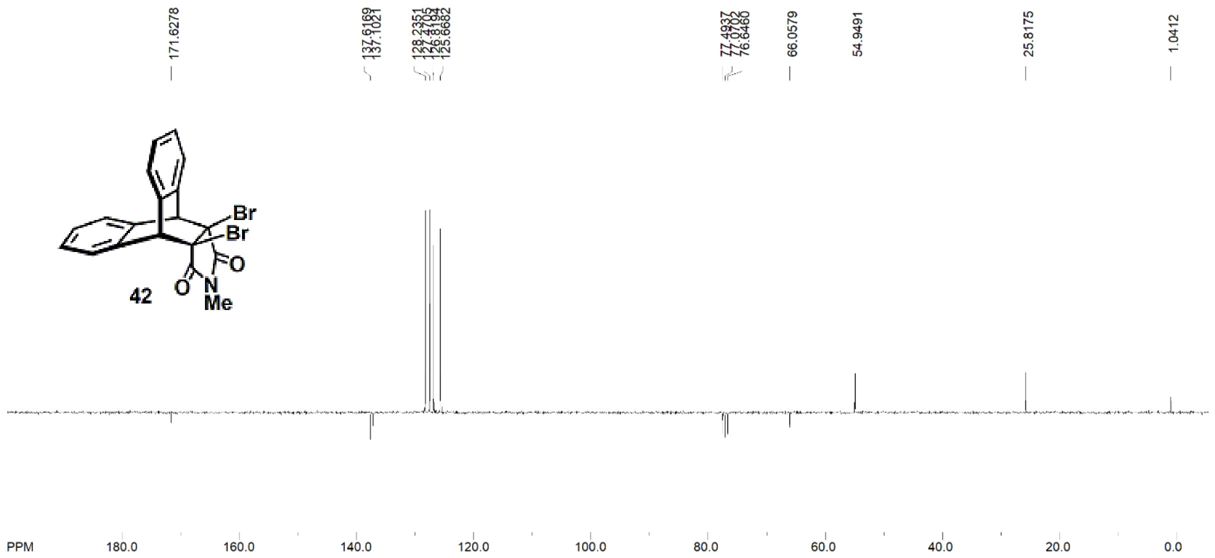

S6. APT ^{13}C NMR (300 MHz, $CDCl_3$) spectrum of **42**.

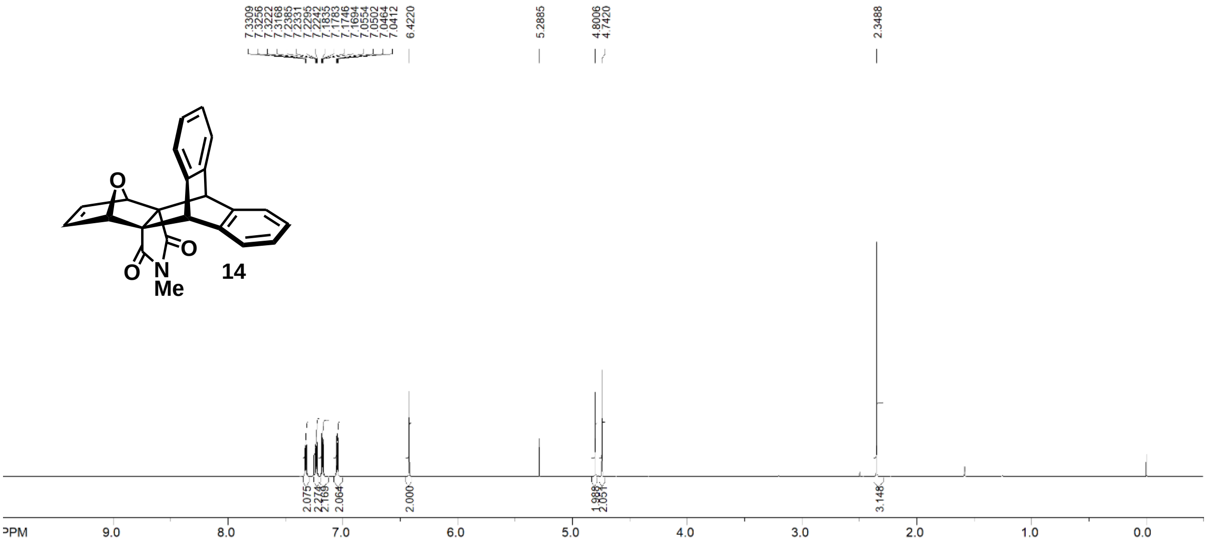

S7. ^{1}H NMR (300 MHz, $CDCl_3$) spectrum of anthracene **14**.

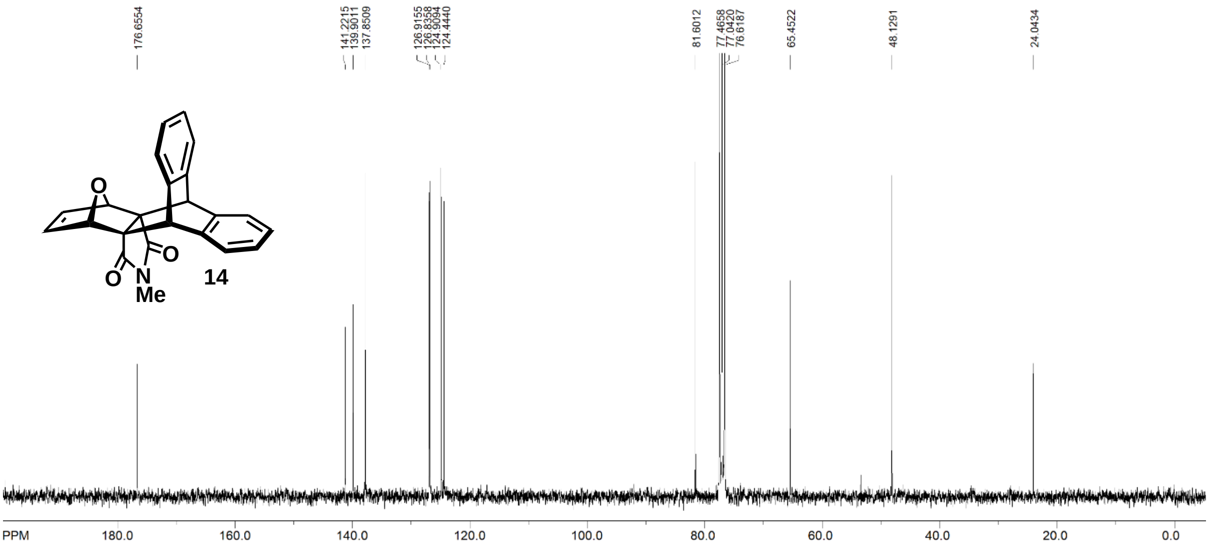

S8. ^{13}C NMR (300 MHz, $CDCl_3$) spectrum of anthracene **14**.

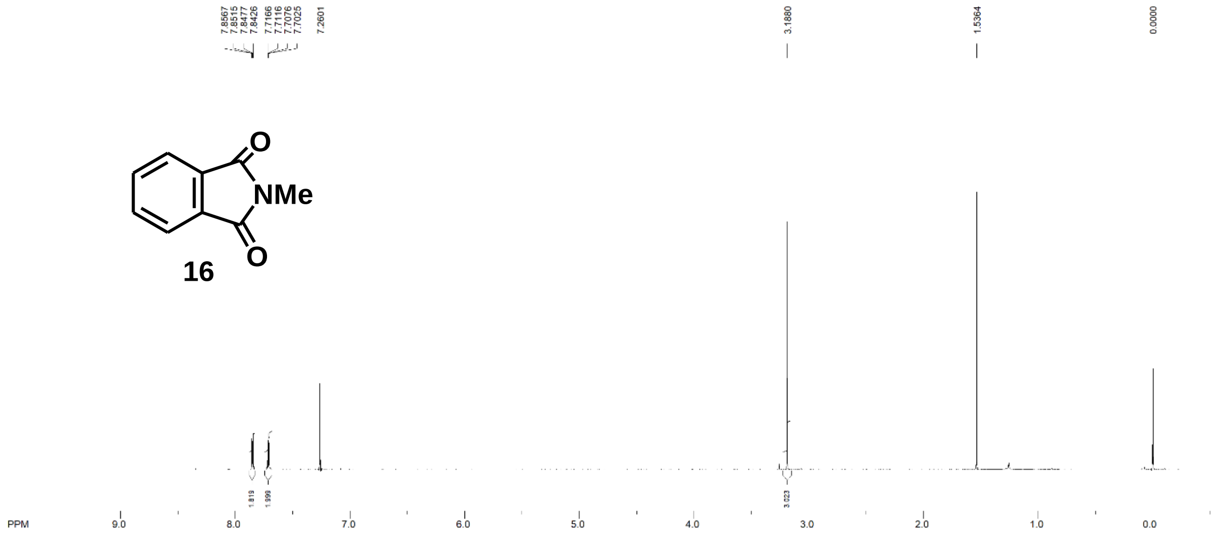

S9. ^{1}H NMR (300 MHz, $CDCl_3$) of -methylphthalimide **16**.

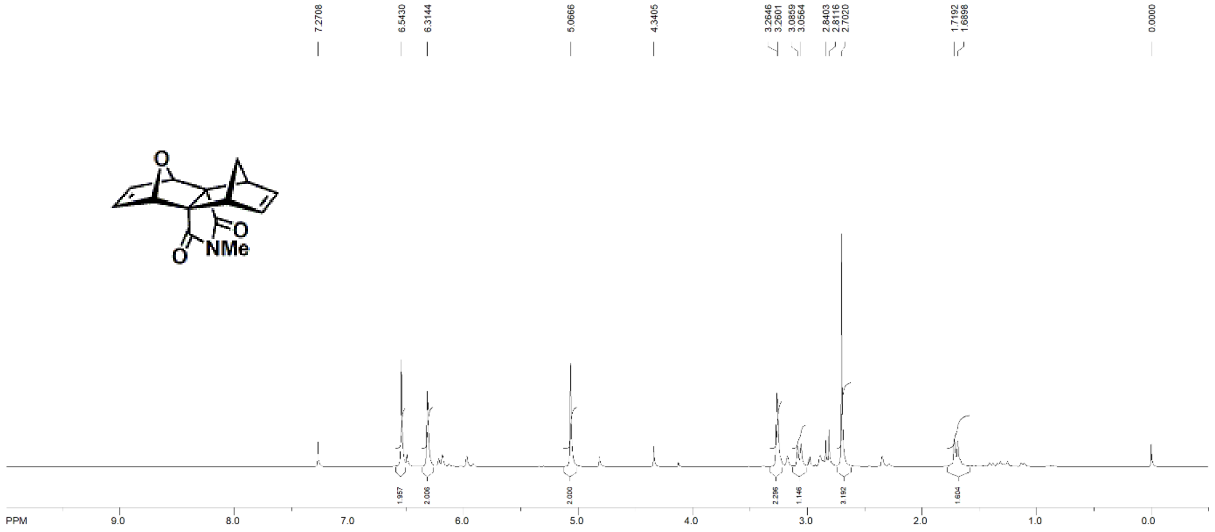

S10. ^{1}H NMR (300 MHz, $CDCl_3$) spectrum of adduct **22**.

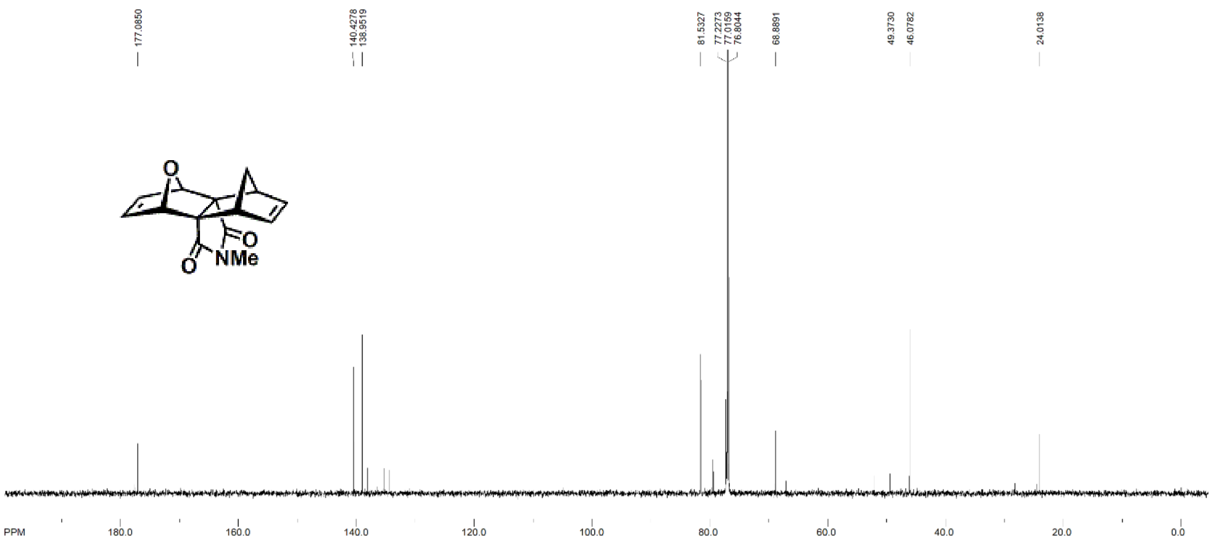

S11. ^{13}C NMR (300 MHz, $CDCl_3$) spectrum of adduct **22**.

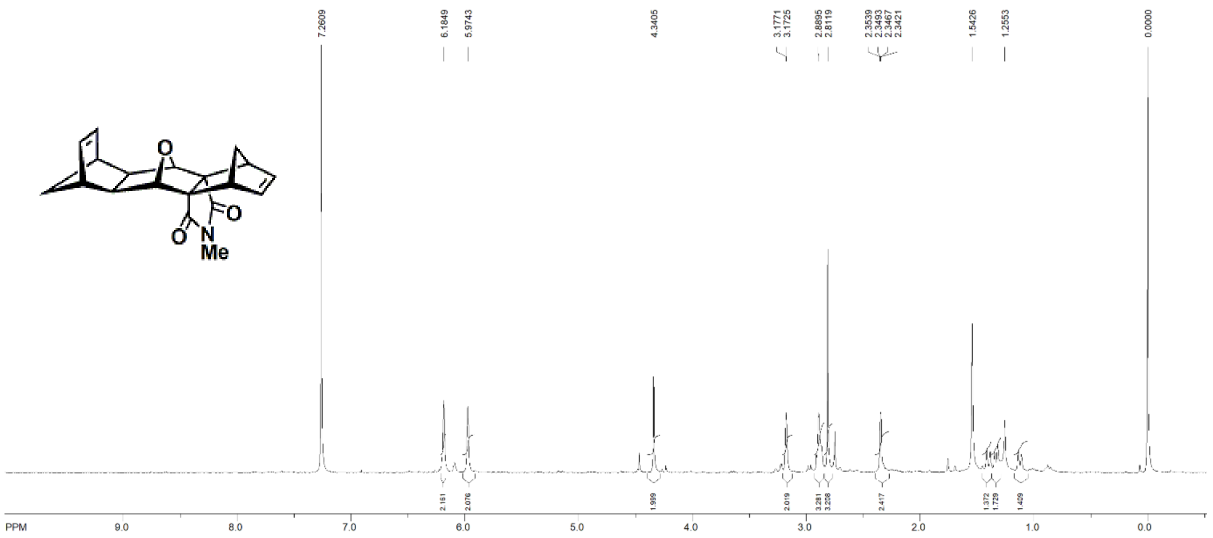

S12. ^{1}H NMR (300 MHz, $CDCl_3$) spectrum of adduct **23**.

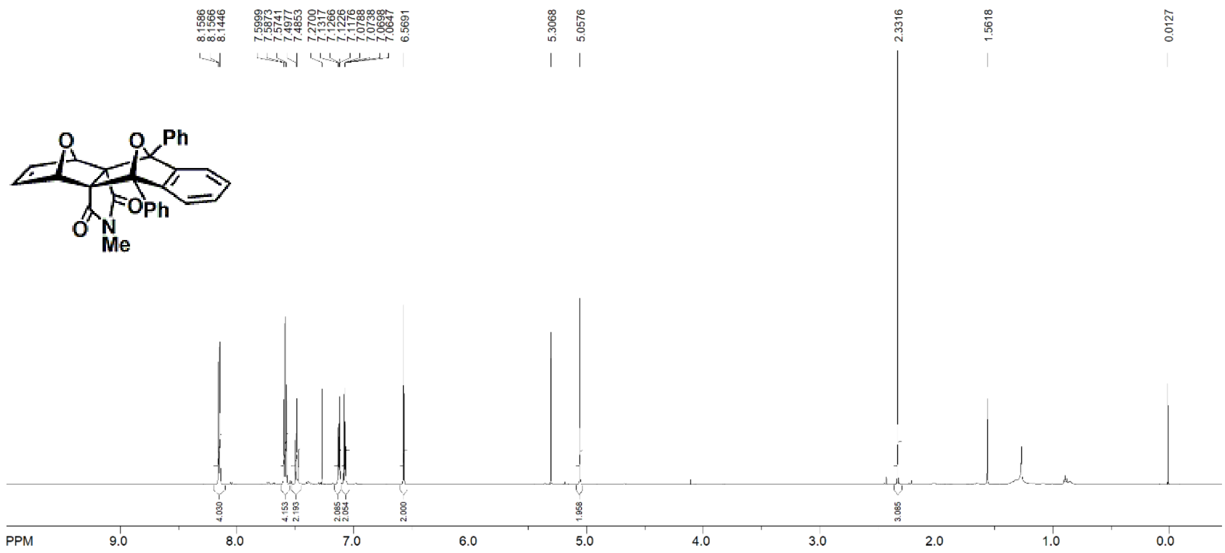

S13. ^{1}H NMR (300 MHz, $CDCl_3$) spectrum of adduct **25**.

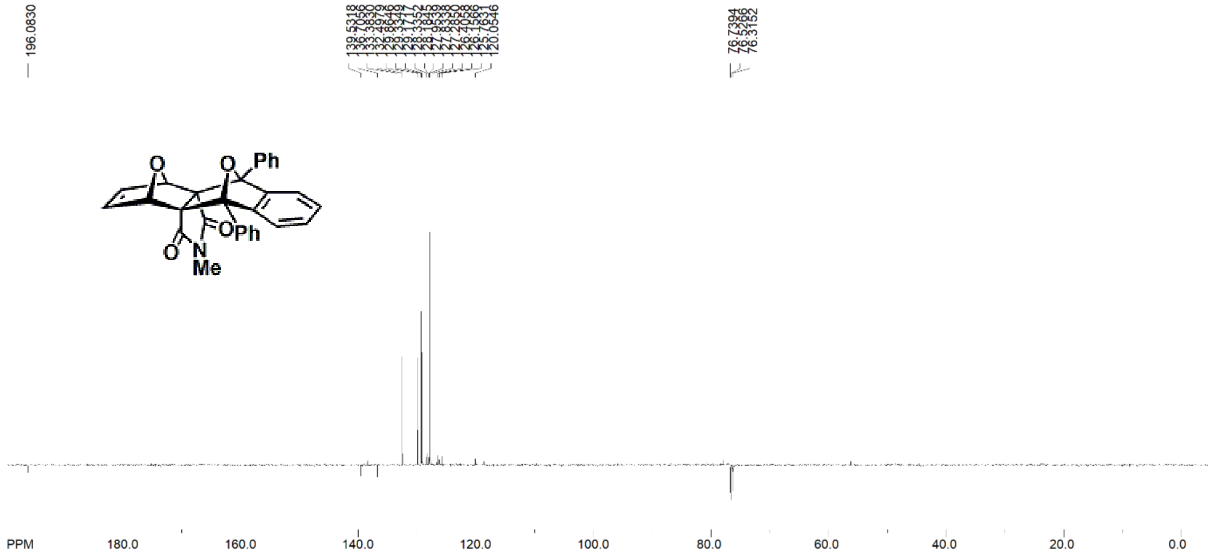

S14. APT ^{13}C NMR (300 MHz, $CDCl_3$) spectrum of adduct **25**.

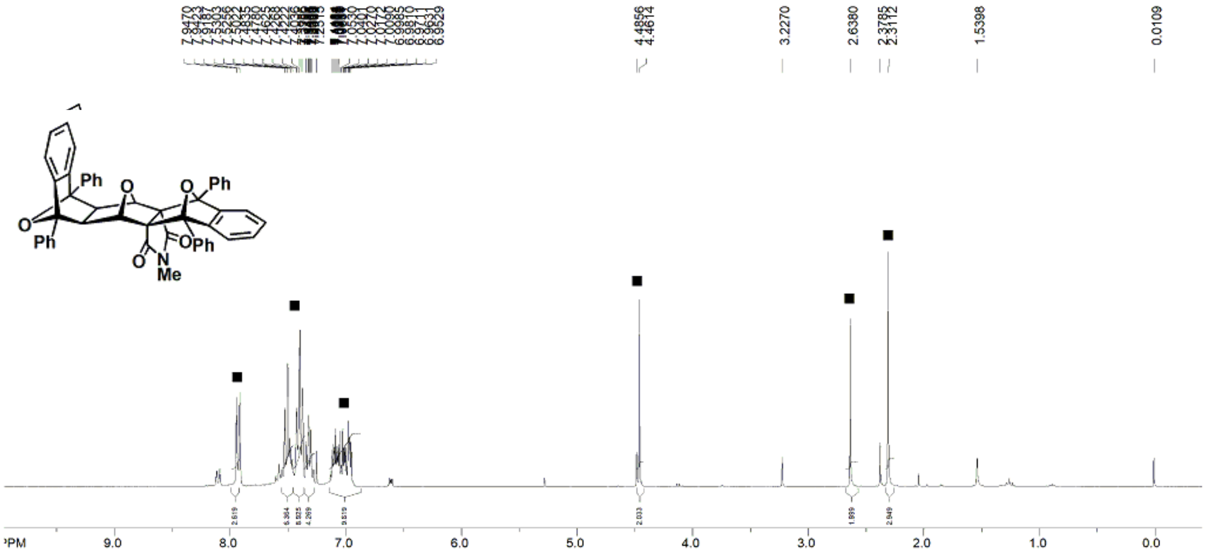

S15. ^{1}H NMR (300 MHz, $CDCl_3$) spectrum of crude adduct **26**.

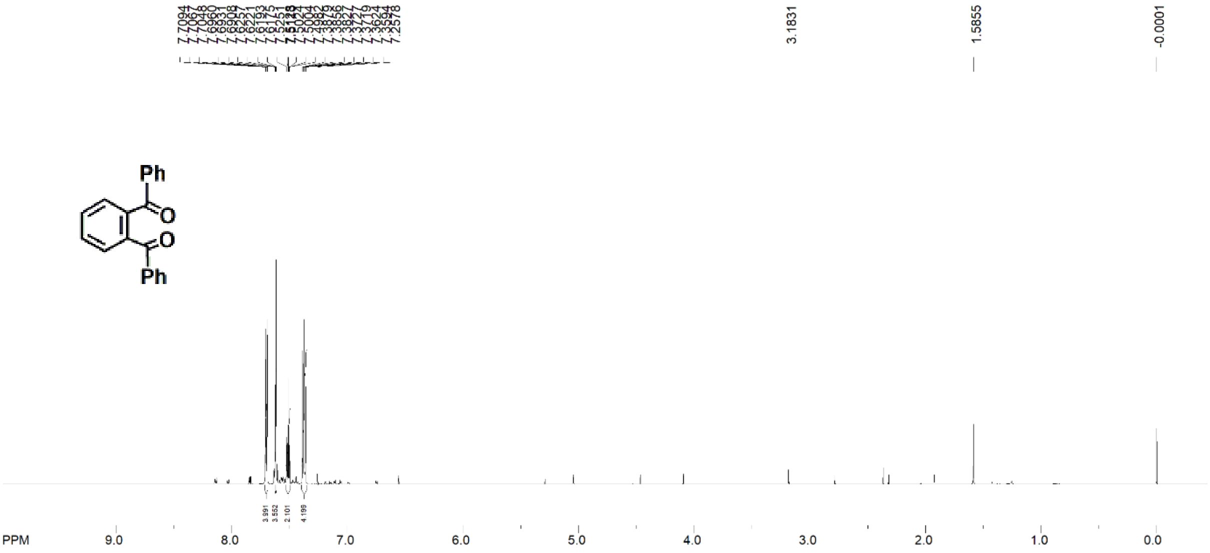

S16. ^{1}H NMR (300 MHz, $CDCl_3$) spectrum of 1,2-dibenzoylbenzene **27**.

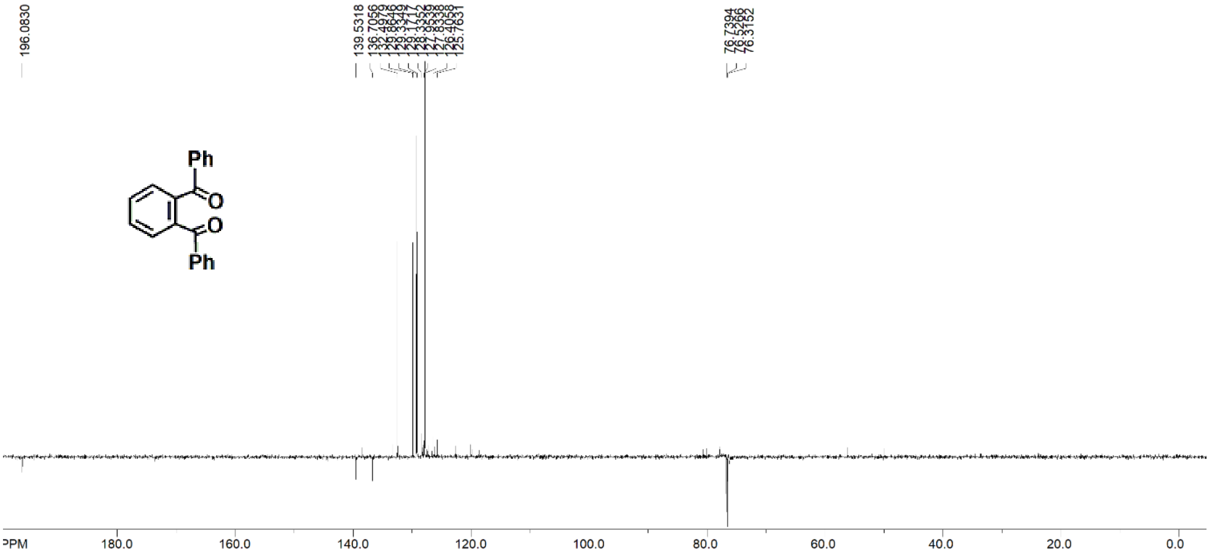

S17. APT ^{13}C NMR (300 MHz, $CDCl_3$) spectrum of 1,2-dibenzoylbenzene **27**.

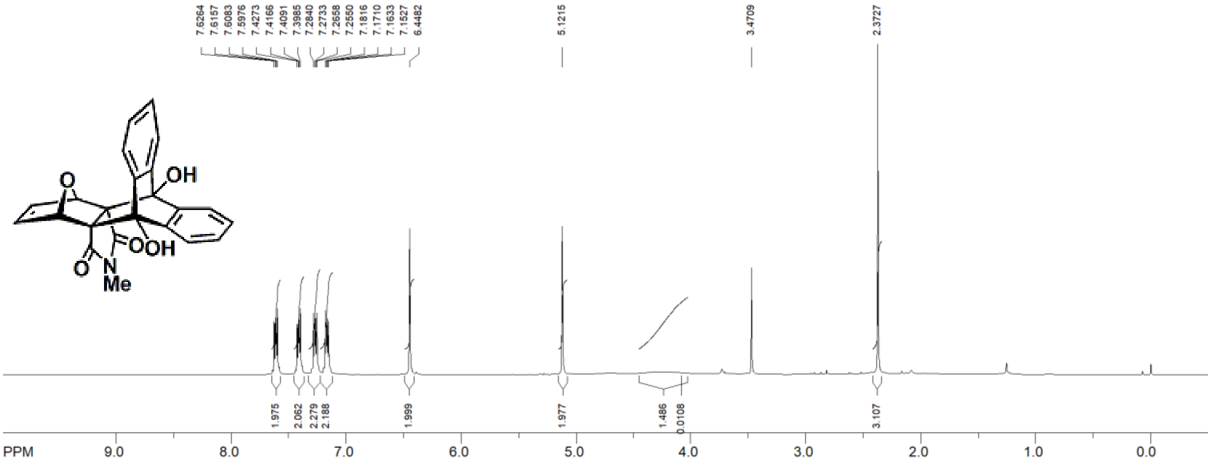

S18. ^{1}H NMR (300 MHz, $CDCl_3$) spectrum of **33**.

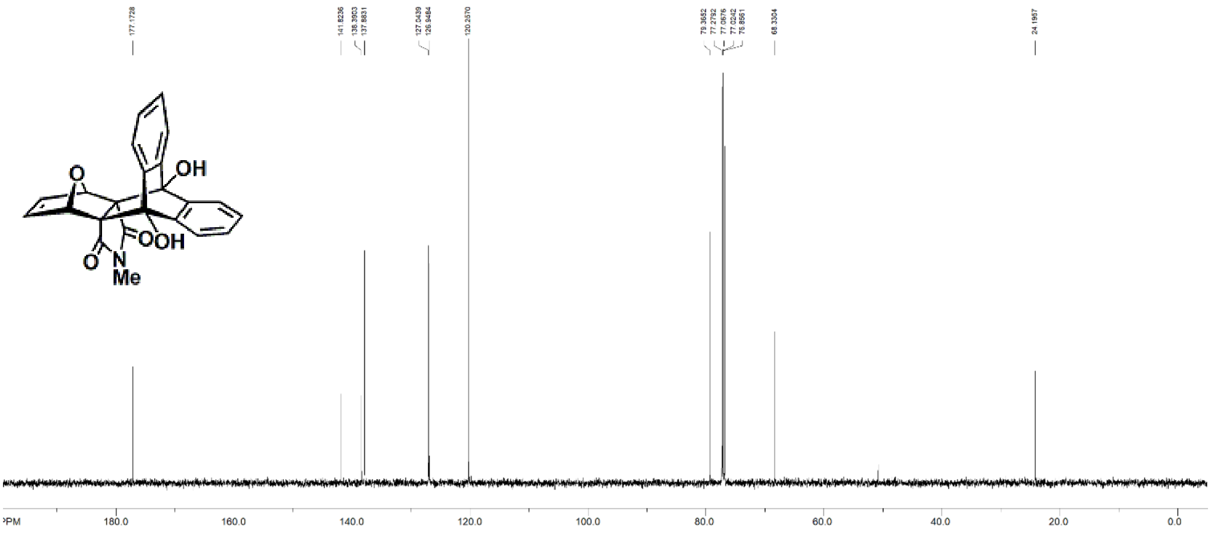

S19. ^{13}C NMR (300 MHz, $CDCl_3$) spectrum of **33**.

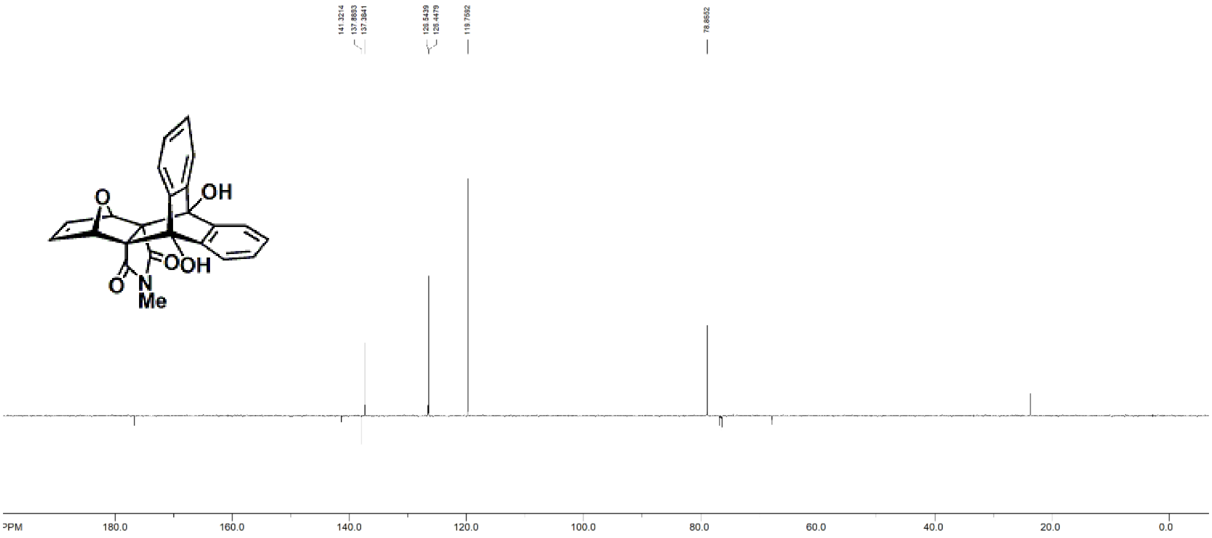

S20. APT ^{13}C NMR (300 MHz, $CDCl_3$) spectrum of **33**.

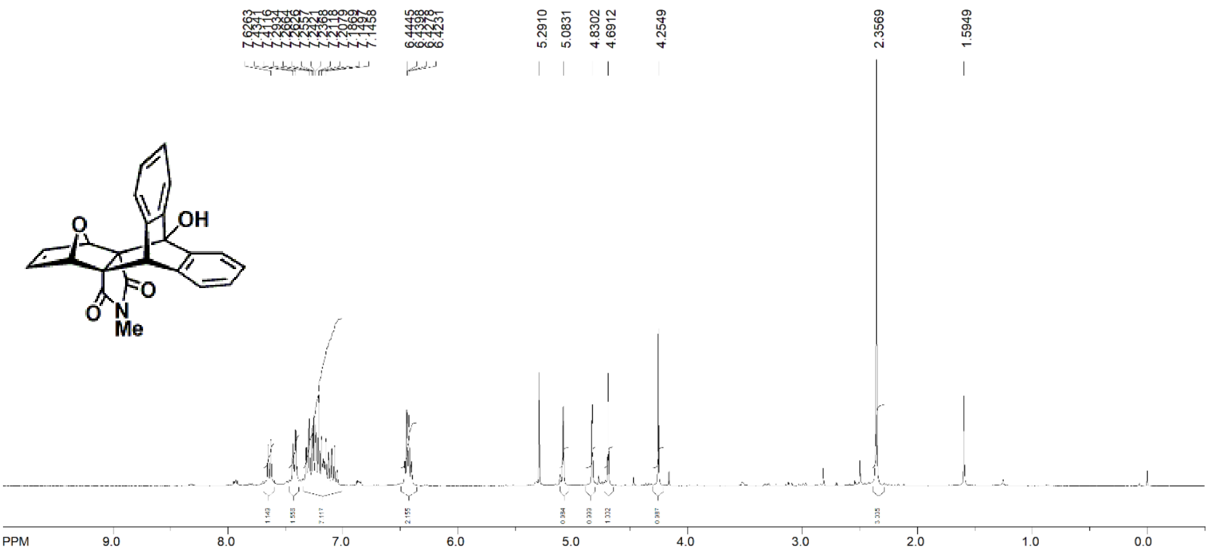

S21. ^{1}H NMR (300 MHz, $CDCl_3$) spectrum of **35**.

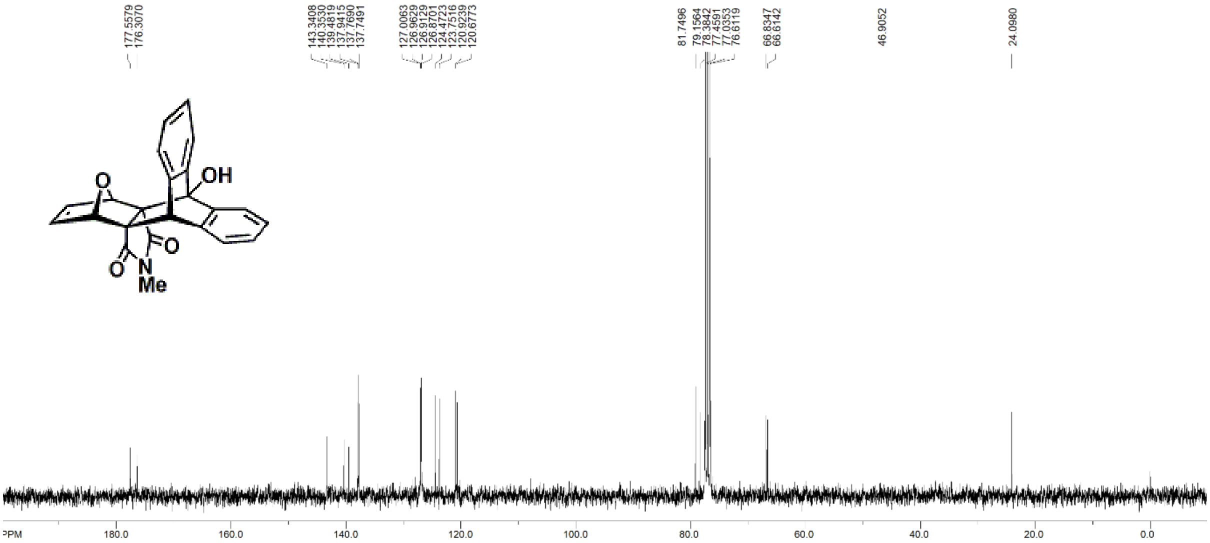

S22. ^{13}C NMR (300 MHz, $CDCl_3$) spectrum of **35**.

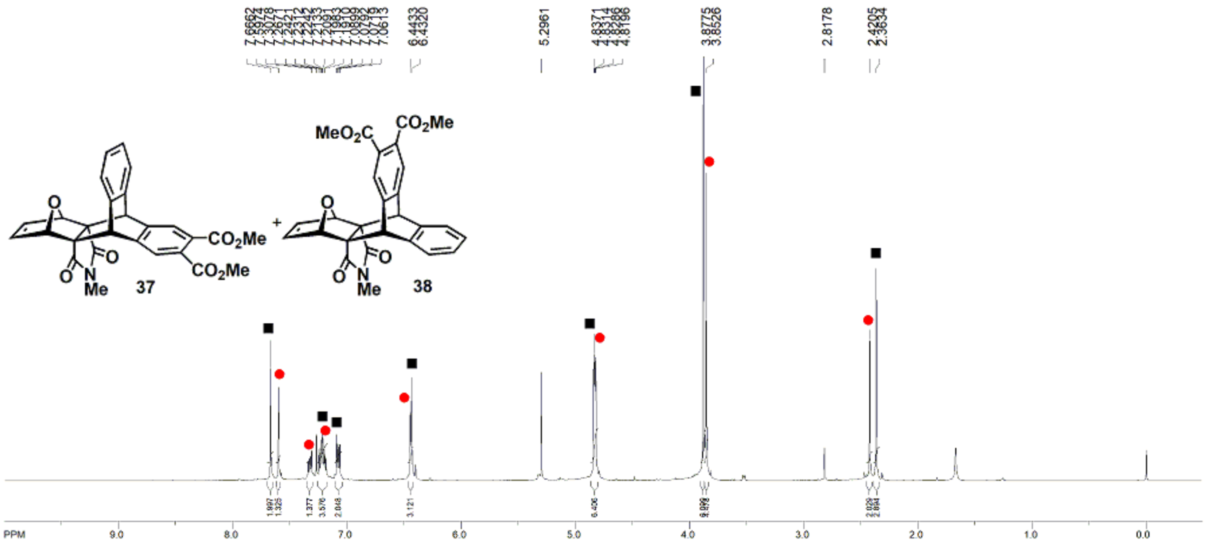

S23. ^{1}H NMR (300 MHz, $CDCl_3$) spectrum of adducts **37** (■) and **38** (•).

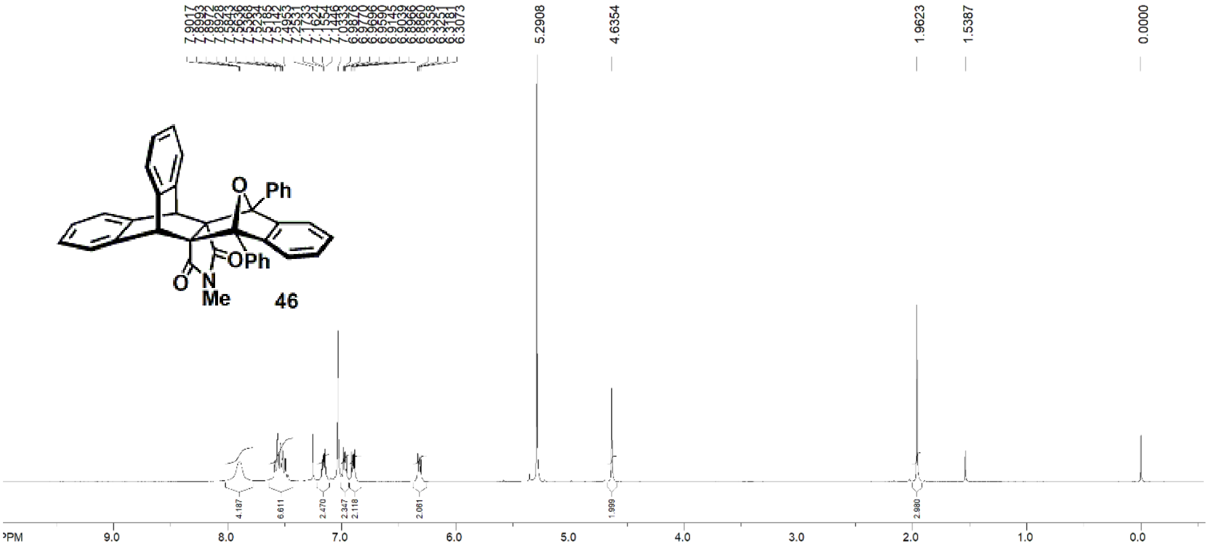

S24. ^{1}H NMR (300 MHz, $CDCl_3$) spectrum of **46**.

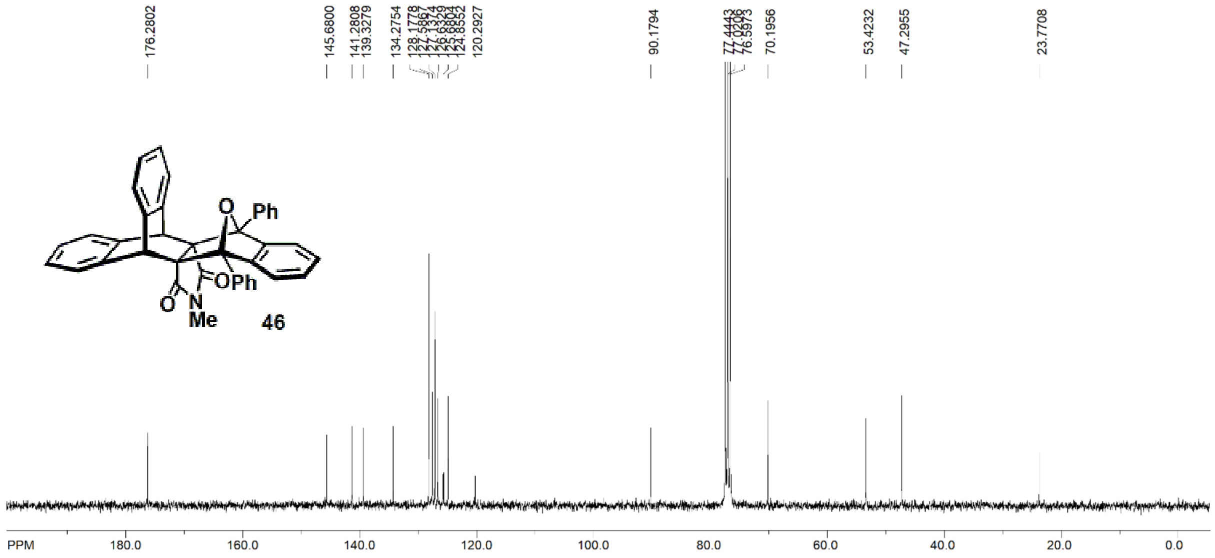

S25. ^{13}C NMR (300 MHz, $CDCl_3$) spectrum of **46**.

4. IR spectra

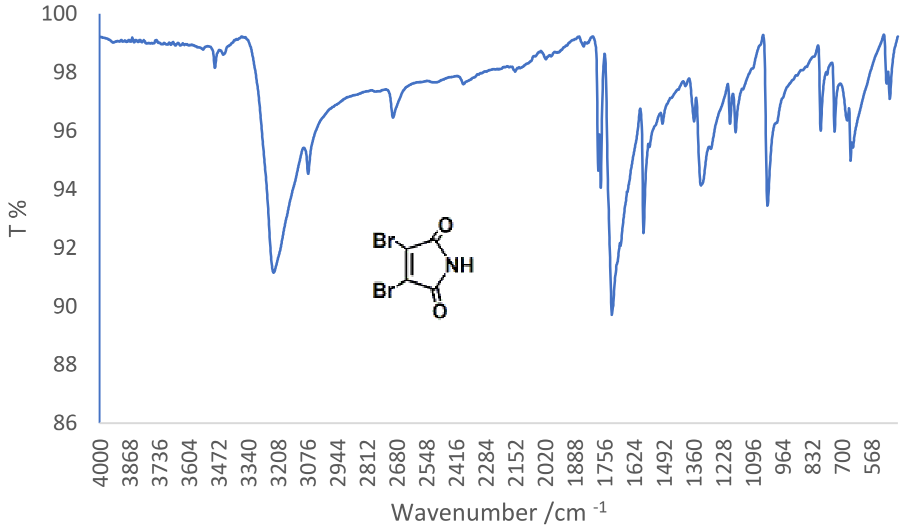

S-IR-1. IR-ATR spectrum of *2,3*-dibromomaleimide.

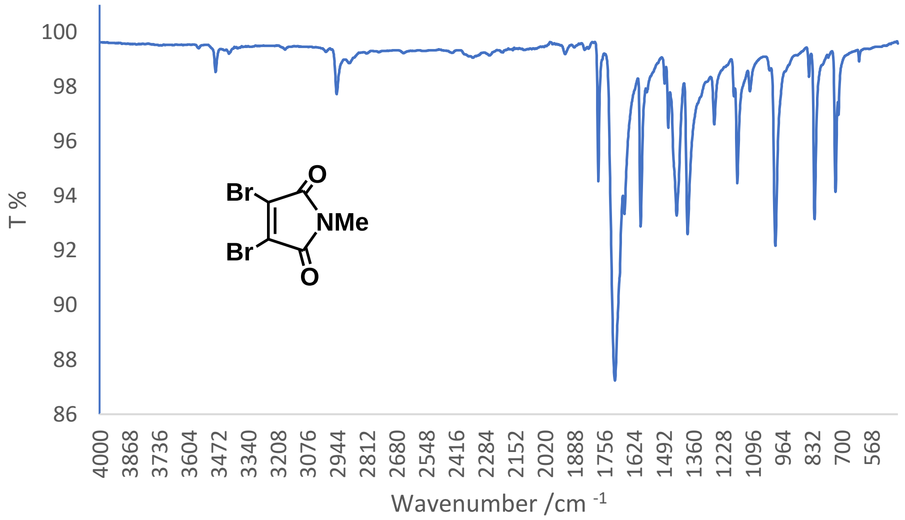

S-IR-2. IR-ATR spectrum of *N*-methyl-2,3-dibromomaleimide.

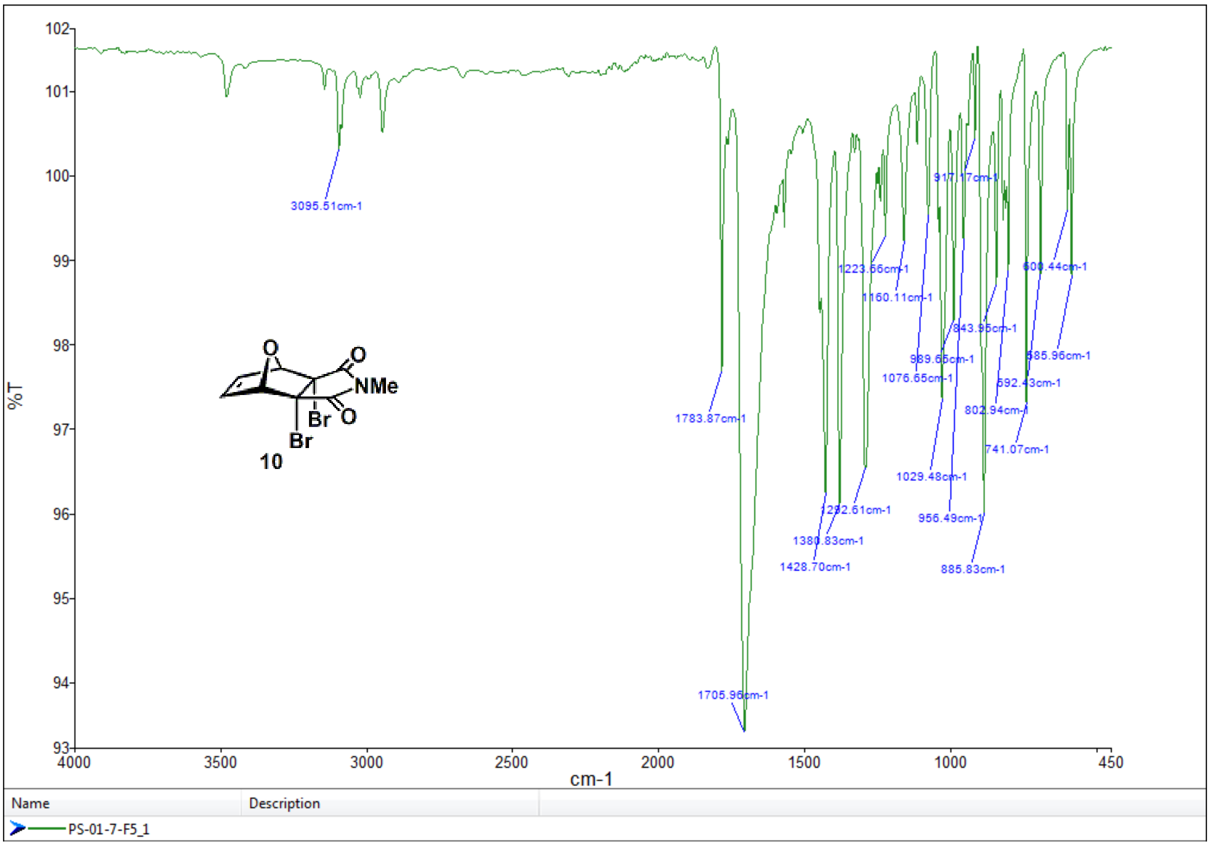

S-IR-3. IR-ATR spectrum of cycloadduct **10**.

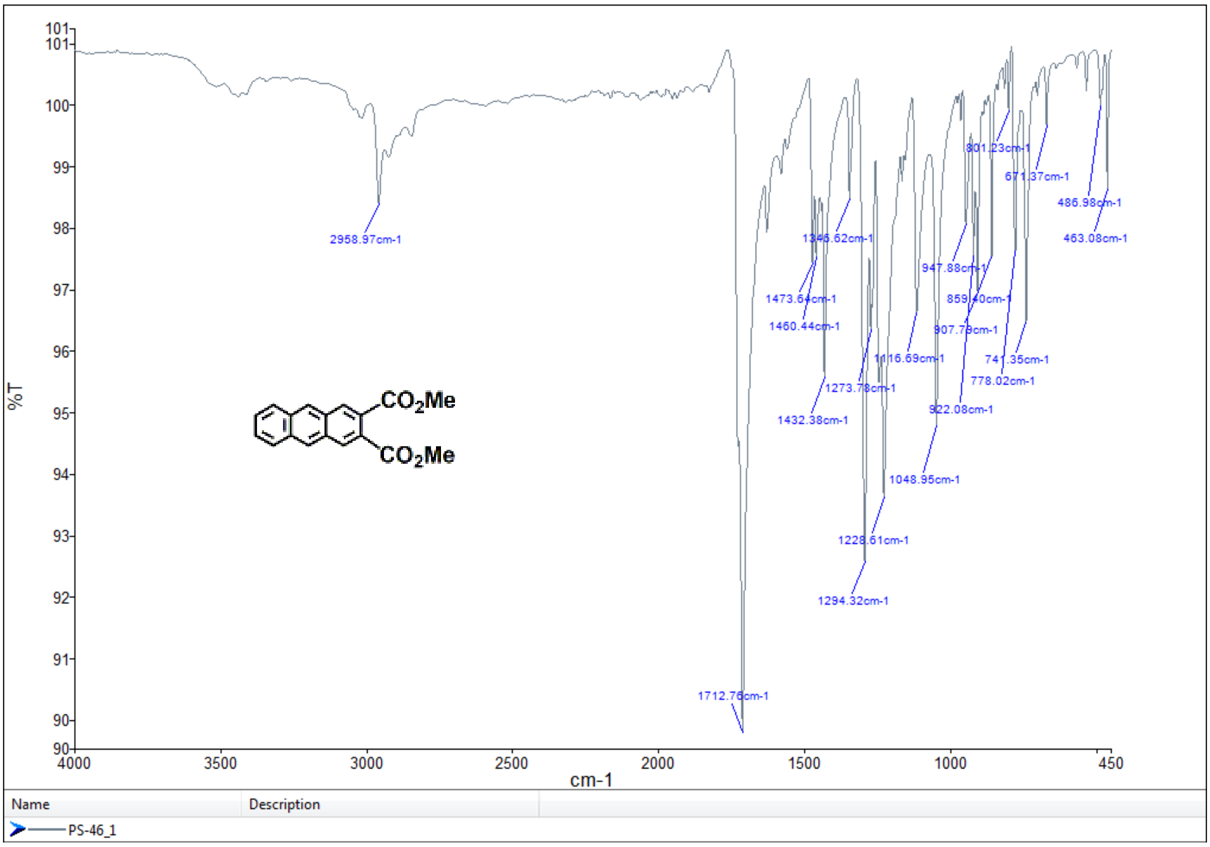

S-IR-4. IR-ATR spectrum of anthracene **36**.

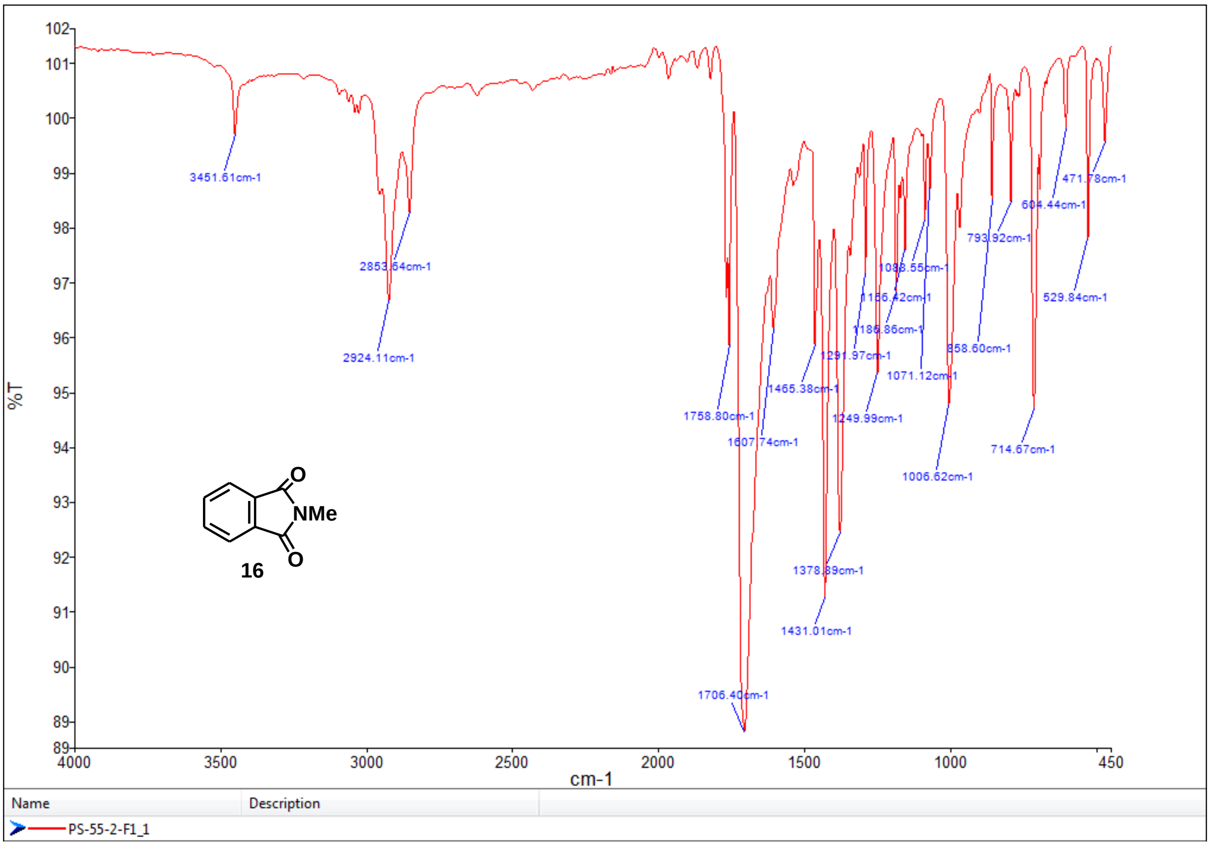

S-IR-5. IR-ATR spectrum of *N*-methylphthalimide **16**.

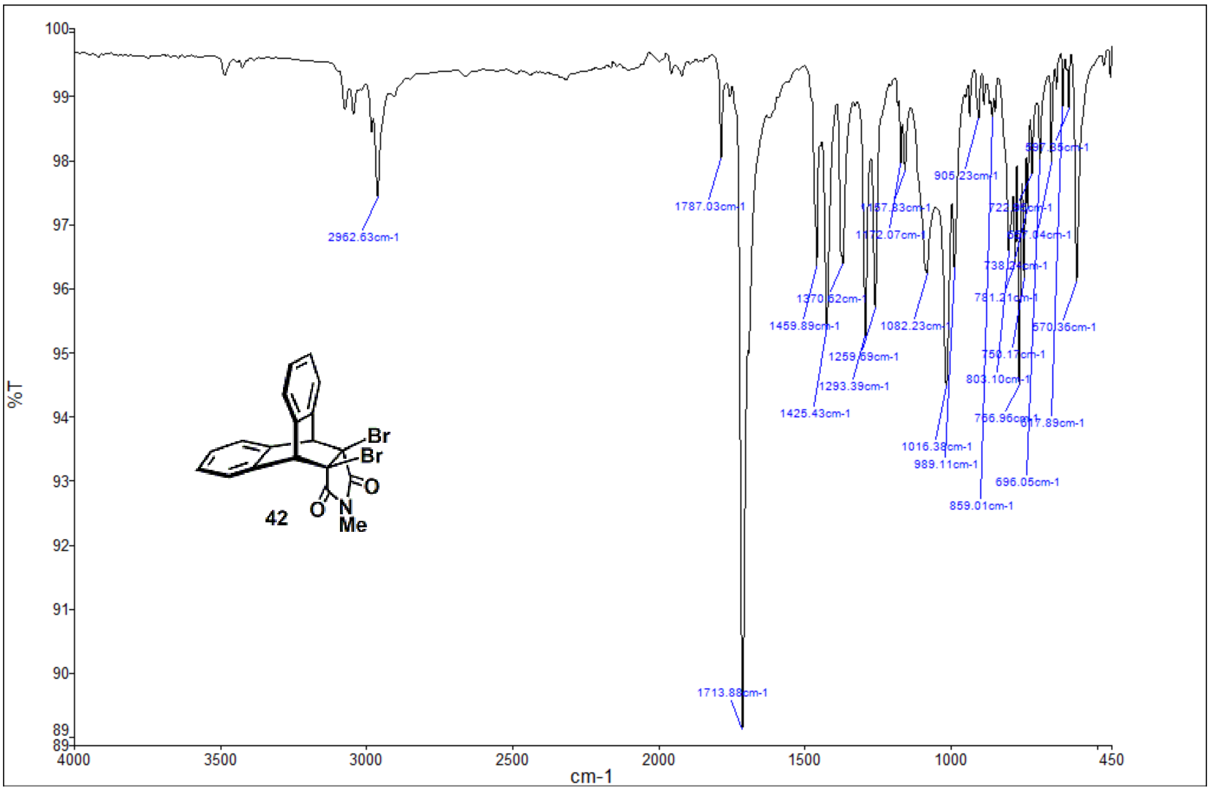

S-IR-6. IR-ATR spectrum of **42**.

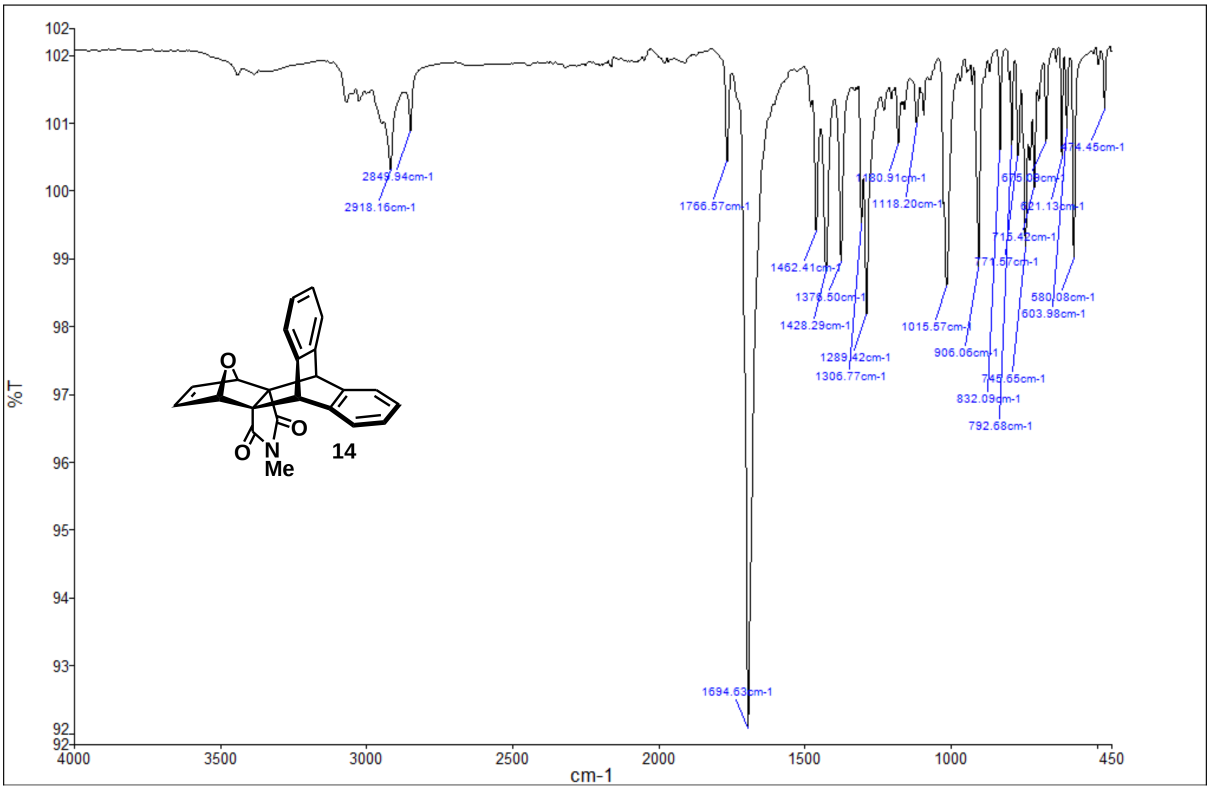

S-IR-47 IR-ATR spectrum of **14**.

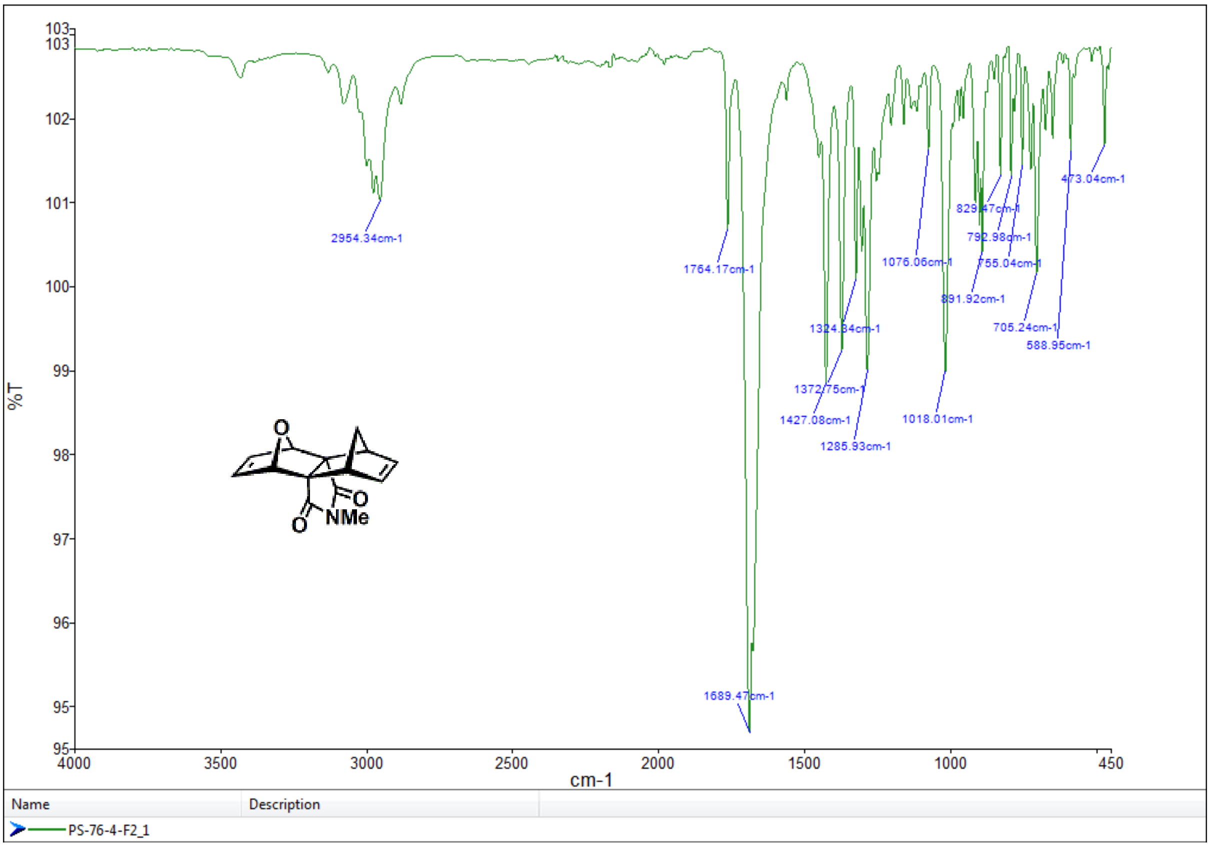

S-IR-8. IR-ATR spectrum of adduct **22**.

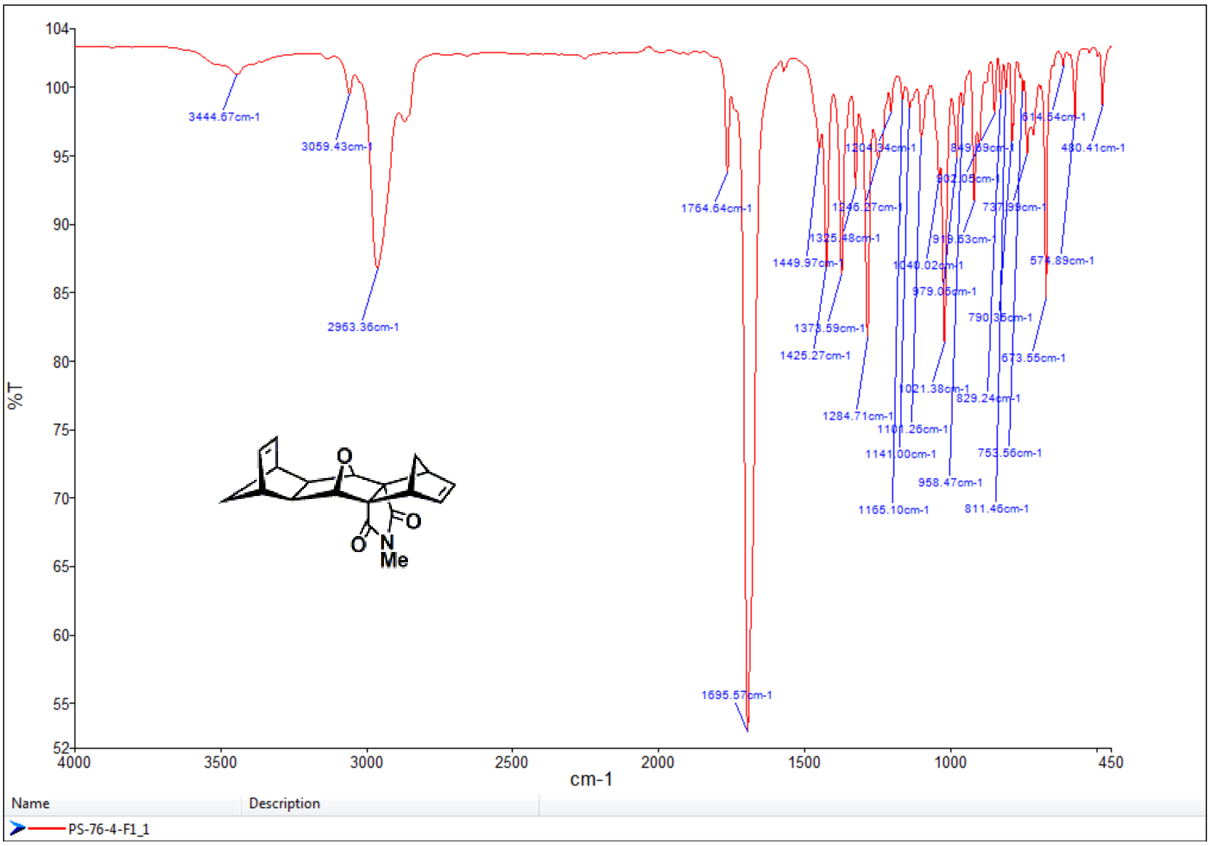

S-IR-9. IR-ATR spectrum of adduct **23**.

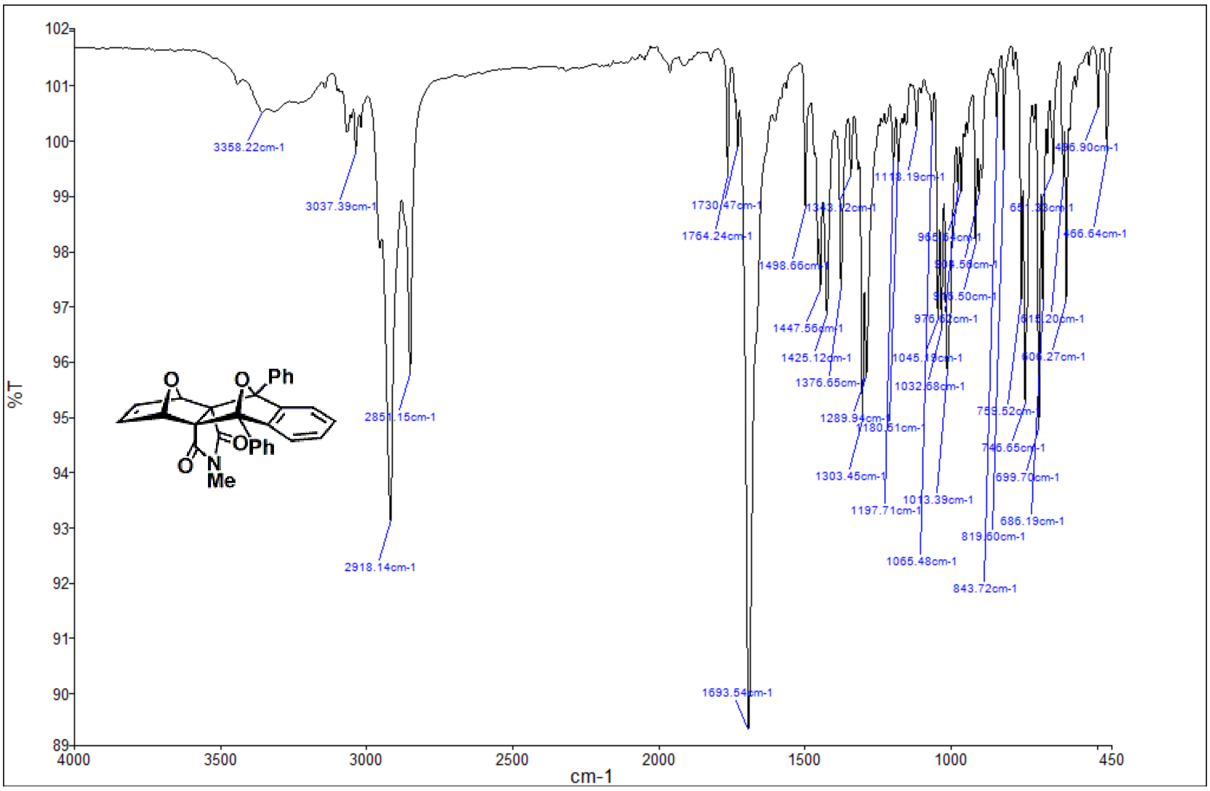

S-IR-10. IR-ATR spectrum of **25**.

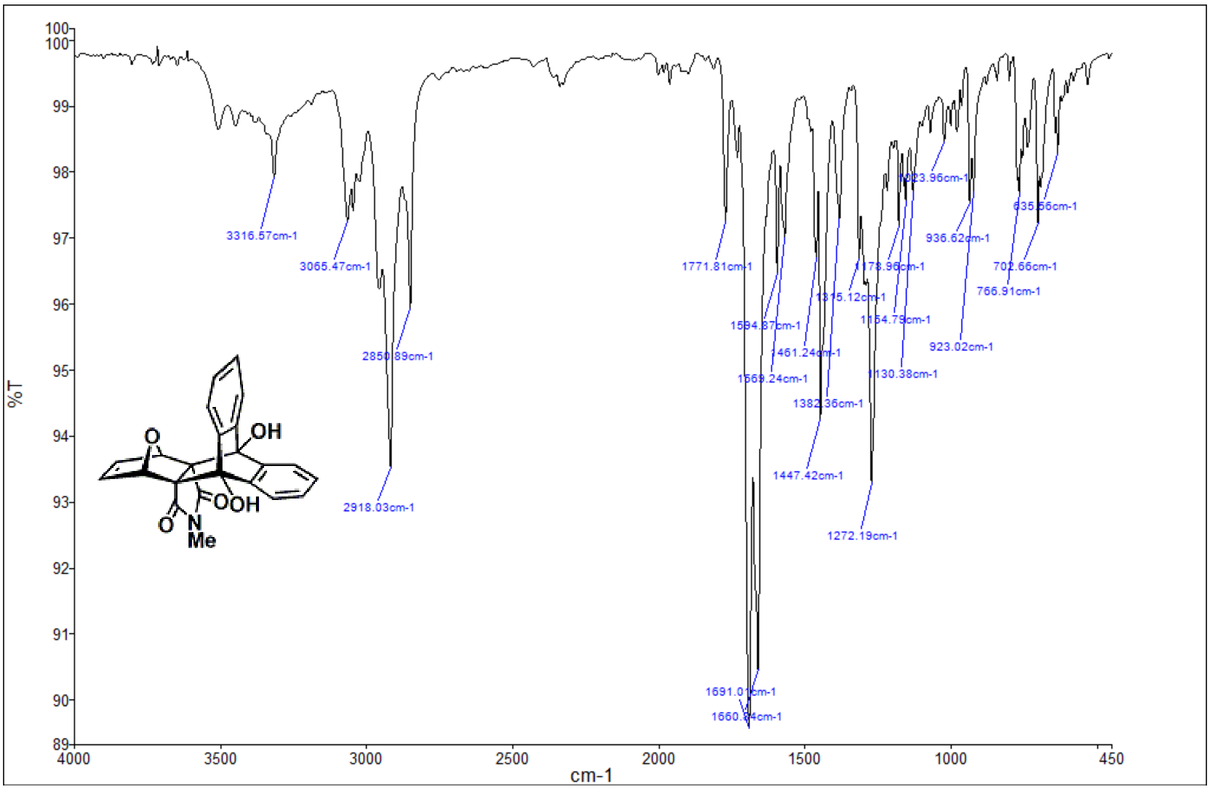

S-IR-11. IR-ATR spectrum of **33**.

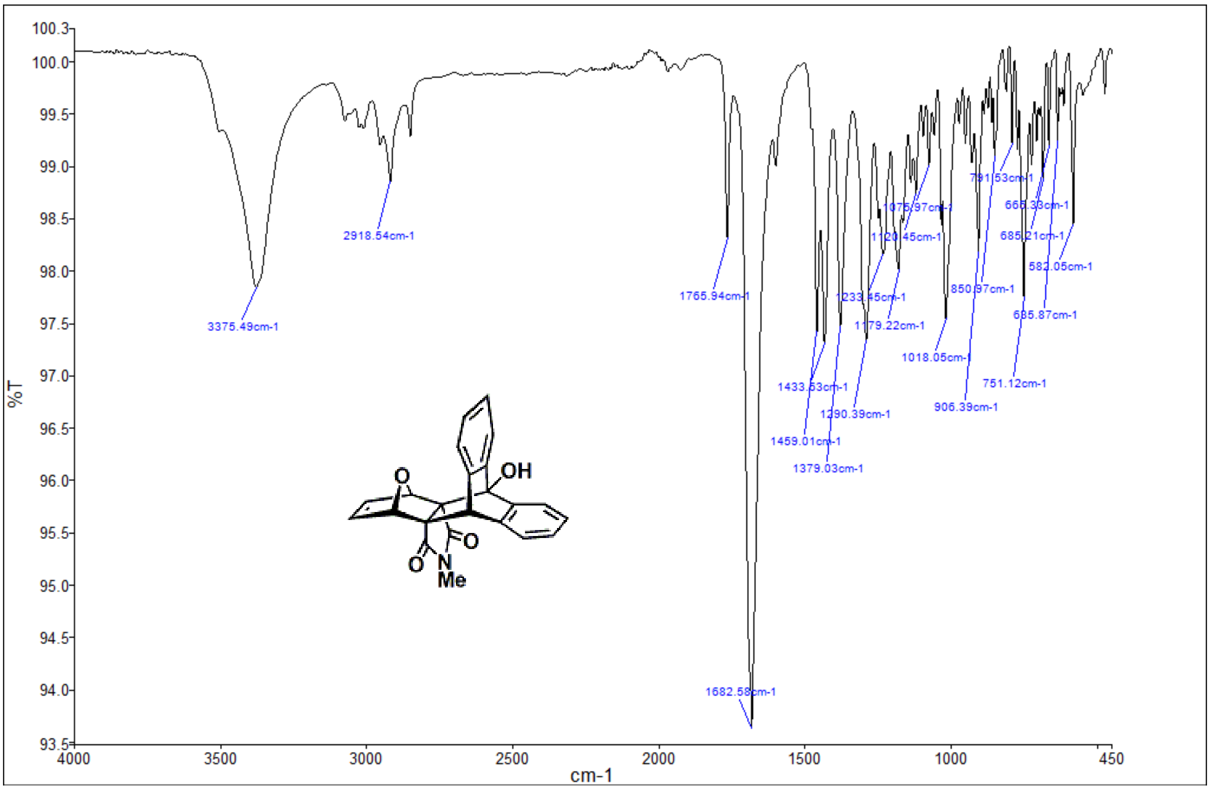

S-IR-12. IR-ATR spectrum of **35**.

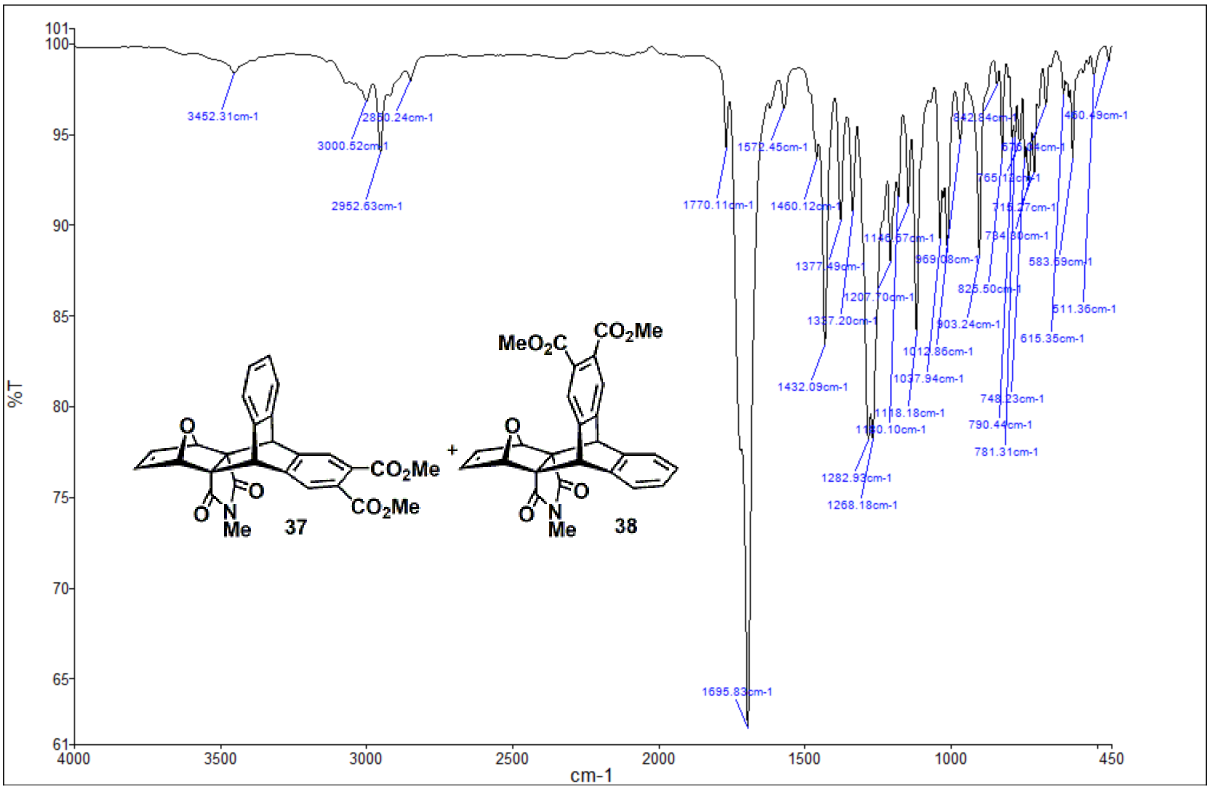

S-IR-13. IR-ATR spectrum of **37** and **38**.

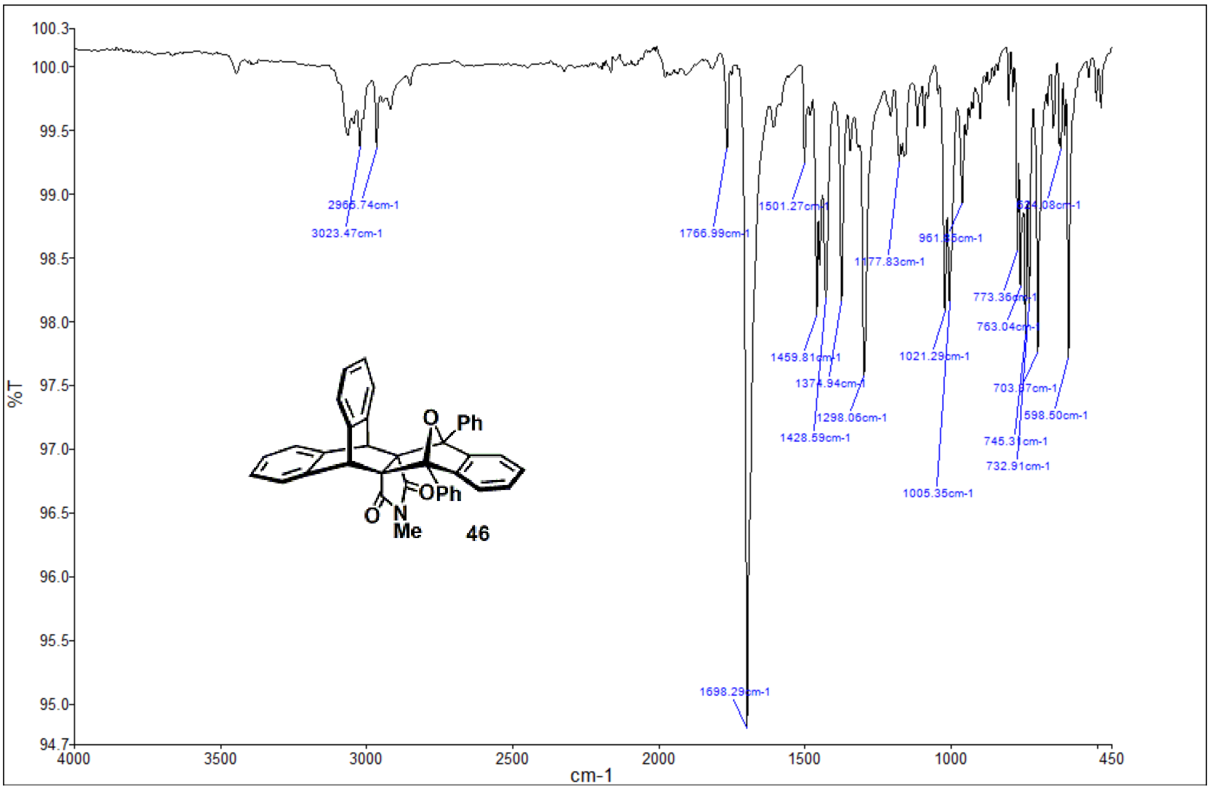

S-IR-14. IR-ATR spectrum of **46**.

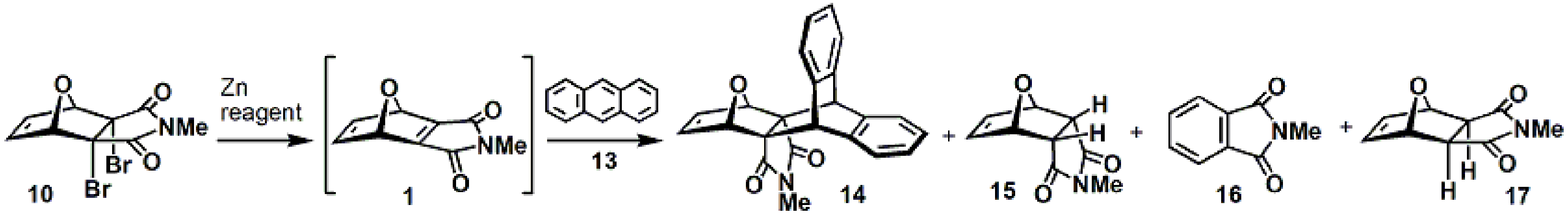

Table S1. Optimization of mechanochemical Zn/Ag debromination of dibromide **10**

**Entry**	**Reducing agent / catalyst**	**Conditions** [a]	**Time/h**	**Conversion /%**	**Yield /%**				**Ratio 10:14:15:16:17**
		**Ball-mill**		**10**	**14**	**15**	**16**	**17**	
**1**	Zn/Ag couple		0.5	81	2	78	1		1:0.11:4.2:0.07
**2**	Zn/Ag couple	NaCl	0.5	quant	8	8	81	3	0:0.1:0.1:1:0.3
**3**	Zn/Ag couple	LAG THF η=1	0.5	quant	58	4	29	9	0:1:0.07:0.5:0.15
**4**	Zn/Ag couple	LAG THF η=1, NaCl	0.5	96	33	13	42	8	0.1:0.8:0.3:1:0.2
**5**	Zn dust, Ag wire		0.5						NR
**6**	Zn dust, Ag wire	LAG THF η=1	0.5	33	27	3	3		1:0.4:0.05:0.05
**7**	Zn dust, Ag wire	LAG MeOH η=1	1	quant	64	9	27		0:0.7:0:1:0.3
**8**	Zn dust, Ag wire	LAG ACN η=1	1	88	63	13	13		0.2:1:0.2:0.2
**9**	Zn activated, Ag wire	LAG THF η=1, NaCl	0.5	quant	56	8	28	8	0:1:0.15:0.5:0.15
**10**	Zn dust, silvergal		0.5						NR
**11**	Zn dust, silvergal	LAG THF η=1	1	50	40	5	5		1:0.8:0.1:0.1
**12**	Zn activated		0.5						NR
**13**	Zn dust		0.5						NR
**14**	Zn dust	LAG THF η=1	0.5	12	4	3	4		1:0.05:0.03:0.05
**15**	Zn dust, Cu dust	LAG THF η=1	0.5	55	50	10	5		0.7:1:0.2:0.1
**16**	Zn dust, Cu dust	LAG THF η=1	0.75	97	64	13	19	1	0.05:1:0.2:0.3:0.02
**17**	Zn dust, Cu dust	LAG THF η=1 $ZnBr_2$	1	quant	67	13	20	0	0:1:0.2:0.3:0
**18**	Zn dust, Cu dust	LAG THF η=0.5	1	66	48	6	10	2	0.7:1:0.2:0.13:0.05
**19**	**Zn dust, Cu dust**	**LAG THF η=0.5**	**2**	**quant**	**76**	**8**	**15**	**0**	**0:1:0.11:0.2:0**
	**Without anthracene**	**DEBROMINATION**							

	Zn dust, Ag wire	LAG THF η=1	0.5	43		29	11	3	1:x:0.5:0.2:0.05+unsym prods
	Zn dust, Ag wire	LAG THF η=1	1	55		45	5	5	1:x:1:0.11:0.1+unsym prods
	Zn dust, Ag wire	LAG MeOH η=1	0.5	95.5		2	75	19	0.06:x:0.03:1:0.25+unsym prods
		**SOLUTION**							
**20**	Zn/Ag couple	dry THF, Ar	1	quant	86	9	5		0:1:0.1:0.06
**21**	Zn dust, Cu dust	dry THF	1						NR
**22**	Zn dust, Cu dust	dry THF,ultrasound	1.5						NR

50 mg dibromide; 132 mg anthracene (5 eq.); 75 mg reducing agent / catalyst; Silvergal = Ag/Cu powder 70% Ag

a) HSVM 30 Hz; LAG THF in η = $\mu L mg^{-1}$, b) 32 mg anthracene (1.2 eq.

5. References

1. Schmidt, E. K. G. *Chem. Ber,* **1974**, *107*, 2440-2452; Ciumician, G. L.; Silber, P. *Chem. Ber,* **1884**, *17*, 553-559. 2,3-dibromomaleimide
2. Scharf, K. *Chem. Ber.* **1965**, 98, 764-780. *N* methyl-2,3-dibromomaleimide
3. Warrener, R. N.; Maksimovic, Lj. *Tetrahedron Lett.* **1994**, *35*, 2389-2392.
4. Lohier, J.-F.; Wright, K.; Peggion, C.; Formaggio, F.; Toniolo, C.; Wakselman, M.; Mazaleyrat, J.-P. *Tetrahedron* **2006**, *62*, 6203-6213. anthracene 2,3-diester
5. Clark, R. D.; Heathcock, C. H. *J. Org Chem.* **1976**, *41*, 636-643. Zn/Ag couple preparation
6. Roux, M. V.; Jiménez, P.; Martín-Luengo, M. A.; Dávalos, J. Z.; Sun, Z.; Hosmane, R. S.; Liebman, J. F. *J. Org. Chem.* 1997, 62, 2732-2737. NMe phthalimide
7. Warrener, R. N.; Elsey, G. M.; Maksimovic, Lj.; Johnston, M. R. *Tetrahedron Lett.* **1995**, *36*, 7753-7756. only chemical shift for NMe signal is reported
8. Yang, L.; Wang, J.; Wang, Y.; Li, X.; Liu, W.; Zhang, Z.; Xie, X. *J. Org. Chem.* **2021**, *86*, 14311-14320.
